# A Comprehensive Study on *Lathyrus tuberosus* L.: Insights into Phytochemical Composition, Antimicrobial Activity, Antioxidant Capacity, Cytotoxic, and Cell Migration Effects

**DOI:** 10.3390/plants13020232

**Published:** 2024-01-14

**Authors:** Rita Jakabfi-Csepregi, Ágnes Alberti, Csenge Anna Felegyi-Tóth, Tamás Kőszegi, Szilvia Czigle, Nóra Papp

**Affiliations:** 1 Department of Laboratory Medicine, Medical School, University of Pécs, Ifjúság u. 13, HU-7624 Pécs, Hungary; csrtaat.pte@tr.pte.hu (R.J.-C.); koszegi.tamas@pte.hu (T.K.); 2 János Szentágothai Research Center, University of Pécs, Ifjúság u. 20, HU-7624 Pécs, Hungary; 3 Department of Pharmacognosy, Faculty of Pharmacy, Semmelweis University, Üllői út 26, HU-1085 Budapest, Hungary; alberti.agnes@semmelweis.hu (Á.A.); felegyi.toth.csenge.anna@semmelweis.hu (C.A.F.-T.); 4 Department of Pharmacognosy and Botany, Faculty of Pharmacy, Comenius University Bratislava, Odbojárov 10, SK-832 32 Bratislava, Slovakia; 5 Department of Pharmacognosy, Faculty of Pharmacy, University of Pécs, Rókus u. 2., HU-7624 Pécs, Hungary; nora4595@gamma.ttk.pte.hu

**Keywords:** *Lathyrus tuberosus*, UHPLC-MS/MS, antimicrobial activity, antioxidant capacity, cytotoxicity, fibroblasts, keratinocytes, cell migration

## Abstract

In this study, in vitro antioxidant, antimicrobial, cytotoxic, and cell migration effects of phenolic compounds of *Lathyrus tuberosus* leaves, known in the Transylvanian ethnomedicine, were investigated. Ultra-high-performance liquid chromatography-tandem mass spectrometry was employed for the analysis of the ethanolic and aqueous extracts. The antimicrobial properties were determined using a conventional microdilution technique. Total antioxidant capacity techniques were used using cell-free methods and cell-based investigations. Cytotoxic effects were conducted on 3T3 mouse fibroblasts and HaCaT human keratinocytes using a multiparametric method, assessing intracellular ATP, total nucleic acid, and protein levels. Cell migration was visualized by phase-contrast microscopy, employing conventional culture inserts to make cell-free areas. Together, 93 polyphenolic and monoterpenoid compounds were characterized, including flavonoid glycosides, lignans, hydroxycinnamic acid, and hydroxybenzoic acid derivatives, as well as iridoids and secoiridoids. The ethanolic extract showed high antioxidant capacity and strong antimicrobial activity against *Bacillus subtilis* (MIC_80_ value: 354.37 ± 4.58 µg/mL) and *Streptococcus pyogenes* (MIC_80_ value: 488.89 ± 4.75 µg/mL). The abundance of phenolic compounds and the results of biological tests indicate the potential for *L. tuberosus* to serve as reservoirs of bioactive compounds and to be used in the development of novel nutraceuticals.

## 1. Introduction

Throughout the documented ages, plants and plant-based products have played a crucial role in human life, serving both as essential foods and therapeutic agents as well. Since 2000, plant-based compounds have gained considerable popularity as valuable repositories of natural remedies, largely due to the high costs and numerous side effects associated with synthetic medicinal compounds [1]. In particular, plant secondary metabolites, including phenolics and flavonoids, exhibit significant biological activities such as antioxidant, antimicrobial, anticancer, and anti-mutagenic properties, all with the slightest detrimental effects on human health compared to their synthetic counterparts [2]. As a result, numerous researchers have redirected their attention to characterizing and investigating the biological effects of plants or their secondary metabolites [3,4]. Due to intense research activity, investigating untapped wild plants within various folk medicine systems shows high potential as a valuable reservoir of biologically active compounds for the development of drugs and functional foods.

Medicinal plants play an important role in ethnomedicine worldwide, as well as in our study carried out in Transylvania, part of Romania. Based on earlier published ethnomedicinal data, including our field studies from 2007 to 2019 in various regions of the country [5,6,7], *Lathyrus tuberosus* L. (tuberous pea, Fabaceae) was selected in our work for further analysis. *L. tuberosus* occurs on road edges, crop fields, and dry grasses in Europe, except in the north and extreme south [8,9].

In various regions of Transylvania, it is known under the vernacular names *tsunya* [10], *borsóvirág*, *vadborsó*, *unalomvirág* [11], *borsó viloja* [12], *disznópityóka* [13], *juliszta*, *csunya*, and *csunyavirág* [5,6]. *Lathyrus* species serve dual purposes, serving as both a food source and as traditional medicines. The tuber of the plant was documented in Renaissance herbals in Switzerland [14], while ethnomedicinally, it was used in Romania in love char, for wounds and stomach aches as a bath, also for sweetness, consumed primarily by children [6,11,12,13,15,16], for digestive problems in Bosnia and Herzegovina [17], and as a snack in Kosovo [18]. Furthermore, *Lathyrus* species play a pivotal role in traditional medicine, exhibiting analgesic properties through the seeds of *L. sativus* L., with anti-inflammatory attributes through aerial components of *L. cicero* L., and efficacy against rheumatism attributed to the leaves of *L. rotundifolius* Willd., particularly within the Turkish pharmacological context [19].

Earlier investigations of *Lathyrus* species have documented the existence of various biologically active compounds, including phenolics, flavonoids, saponins, and polyunsaturated fatty acids [20,21].

To our knowledge, there is no existing study on the biological and chemical profiles of *Lathyrus tuberosus* in scientific databases. Thus, the purpose of this study was to extract the aerial part of *L. tuberosus* to assess its antioxidant, antimicrobial, cytotoxic, and cell migration effects, correlating them with its chemical fingerprints by LC-MS. The results obtained may provide valuable information for the formulation of new (traditional) herbal medicines and functional ingredients. In ethnomedicine, various parts of plants are utilized for medicinal purposes. We used only leaves in our experiments because this can offer several advantages, such as easy accessibility and manageability. Additionally, leaves frequently contain biologically active compounds that may exhibit various biological effects. Therefore, the application of leaves can facilitate the easy control of experimental conditions and the elucidation of properties of interest.

## 2. Results

### 2.1. Qualitative Analysis of Phenolic Compounds in Plant Extracts with UHPLC-DAD-ESI-MS/MS

Ultra-high-performance liquid chromatography-diode array detection coupled to electrospray ionisation tandem mass spectrometry (UHPLC-DAD-ESI-MS/MS) in negative ionisation mode was used to tentatively identify the phenolic profile of the studied *L. tuberosus* extracts. 93 compounds were characterized by comparing their retention times, UV spectra, and mass spectrometric fragmentation with the literature data. The results are presented in Table 1 and Table 2. The UHPLC-UV chromatograms (320 nm) of the extracts are summarised for the ethanolic and aqueous leaf extracts separately in Figures S1 and S2 (Supplementary Materials).

The composition of the aqueous and 50% (*v*/*v*) ethanolic extracts showed only minor differences. Caffeic acid-*O*-hexoside (**1**), some quercetin-*O*-oligoglycosides (e.g., **17**, **34**, **35**), compound **31** (isorhamnetin-*O*-hexosyl-deoxyhexoside), compound **49** (apigenin-7-*O*-sinapoyl-pentoside) and both monoterpene derivatives were present only in the ethanolic extract. On the other hand, quercetin-3-*O*-acetylhexoside (**71**) and kaempferol-3-*O*-deoxyhexoside (**81**) were detected only in the aqueous sample. Compounds **29** (quercetin-3-*O*-deoxyhexosyl-7-*O*-deoxyhexoside) and **33** (coumaroyl-dimethyl-dihydroxybutanedioic acid) were also observed with remarkably higher intensities compared to the ethanolic extract.

#### 2.1.1. Flavonoid Derivatives

- Flavonol-*O*-glycosides

In line with previous studies, numerous flavonol, flavone, and flavanone derivatives were detected in their glycosidic forms, similar to other species of *Lathyrus* [21,22,23]. In the case of *O*-glycosides, neutral losses of 132, 162, 146, 204, and 176 Da indicated the cleavage of a pentose, a hexose, a deoxyhexose, an acetylhexose, or a glucuronic acid moiety, respectively [24]. Fragment ions of flavonoids generated by the loss of sugar units were observed at *m*/*z* 285, 301, and 315, corresponding to deprotonated kaempferol, quercetin, and isorhamnetin aglycones, respectively. In addition to these Y_0_^−^ aglycone ions, abundant radical product ions [Y_0_−H]^−•^ or [M−H−^•^Gly_(3)_]^−•^ formed by homolytic cleavage of the saccharide moiety were also present in the mass spectra of 3-*O*-glycosylated flavonols. Additional [Y_0_−2H]^−^ or [M−H−^•^Gly_(3)_−^•^Gly_(7)_]^−^ ions were detected for glycosylated compounds at C3-OH and C7-OH positions [25] (Table 2). Taking into account these, we assumed that compounds **37**, **42**, and **71** detected at *m*/*z* 463, 463, and 505, are two quercetin-3-*O*-hexoside isomers and quercetin-3-*O*-acetylhexoside, respectively [21,22,26]. Similarly, compound **81**, which shows its [M−H]^−^ ion at *m*/*z* 431, was tentatively characterized as kaempferol-3-*O*-deoxyhexoside [21,26,27]. Compounds **17**, **27**, and **35** presented deprotonated molecular ions at *m*/*z* 609. Based on the presence of the fragment ion [M−H−Gly_(7)_]^−^ or [M−H−146]^−^ at *m*/*z* 463, referring to the loss of a deoxyhexose moiety from position C7, components **17** and **27** were established as quercetin-3-*O*-hexosyl-7-*O*-deoxyhexoside isomers. On the other hand, the [M−H−Gly_(7)_]^−^ ion was missing in the mass spectra of compound **35**, therefore, it was characterized as quercetin-3-*O*-deoxyhexosyl-hexoside [21,26]. Compounds **29**, **44**, and **36** presented fragment ions at *m*/*z* 446, 430, and 460 as well as fragment ions at *m*/*z* 299, 283, and 283, respectively; therefore, these were characterized as quercetin and kaempferol-3,7-di-*O*-glycosides [23,25,28]. Quercetin-3,7-*O*-oligoglycosides were also detected using the same interpretation method: quercetin-3-*O*-deoxyhexosyl-hexosyl-7-*O*-deoxyhexosyl-hexoside (**56**) with [M−H]^−^ ion at *m*/*z* 917, quercetin-3-*O*-deoxyhexosyl-7-*O*-glucuronyl-hexosyl-hexoside (**63**) at *m*/*z 947* and quercetin-3-*O*-hexosyl-7-*O*-hexosyl-hexosyl-deoxyhexoside **41** at *m*/*z* 933 [21]. Although no ions of the diagnostic product were detectable for compound **34**, based on data from the literature, it was assigned as an isomer **41 [21]**. Compound **26** that displayed the [M−H]^−^ ion at *m*/*z* 741 presented the [Y_0_−H]^−•^ product ion corresponding to the radical aglycone at *m*/*z* 300. The position of glycosylation could not be indicated by this single product ion. Therefore, **26** was characterized as quercetin-*O*-deoxyhexosyl-hexosyl-pentoside [23]. Compound **31** at *m*/*z* 623 presented neutral losses of 146 and 162 Da, and a fragment ion at *m*/*z* 315. According to the literature, both the flavonol glycoside isorhamnetin-*O*-hexosyl-*O*-deoxyhexoside and the caffeoyl-phenylethanoid-glycoside verbascoside (or acteoside), presenting the same fragment ions, have been described in *Lathyrus* species [21,22]. The neutral loss of 146 atomic-mass unit (amu) indicates the cleavage of a rhamnose moiety for both potential structures. However, the loss of 162 Da could indicate either the elimination of a hexose or that of a caffeoyl moiety. Therefore, the structure of compound **31** could not be established unambiguously; it was presumed either as isorhamnetin-*O*-hexosyl-*O*-deoxyhexoside or verbascoside.

- Flavonol-*O*-acylglycosides

Compounds **50**, **52**, **53**, **57**, **61**, **64**, **67**–**70**, **72**, **74**, **79**, **82**, **84**–**93** exhibited [M−2H]^2−^ ions with high intensity, while the corresponding [M−H]^−^ ions were observed to be much less abundant. As detailed previously, the analysis of the neutral losses yielded by the cleavage of sugar residues from the flavonol-*O*-glycosides could be employed for their structural characterization. The losses of 132, 162, 146, and 176 Da pointed to the elimination of residues of pentose, hexose, deoxyhexose, and glucuronic acid, respectively. However, neutral losses of 176, 146, and 162 Da could also indicate the cleavage of ferulic acid, coumaric acid, or caffeic acid moieties, respectively. Fortunately, the hydroxycinnamic acid derivatives presented additional characteristic ions that enabled their tentative identification. Fragment ions corresponding to the deprotonated hydroxycinnamic acids were detected at *m*/*z* 179, 163, 223, and 193 for caffeic acid, coumaric acid, sinapic acid, and ferulic acid respectively, while additional typical ions at *m*/*z* 161, 145, 205, and 175 were generated by cleavage of a H_2_O molecule [24]. Furthermore, a neutral loss of 264 Da was also detected in the mass spectra of compounds **72**, **88**, and **91**, indicating the loss of a dihydrophaseoyl moiety. The appearance of esterified kaempferol-*O*-glycosides with dihydrophaseic acid was previously reported as representative of *L. cicera* L. seeds [23]. We also could deduce the position of the saccharide residues based on the results of Ferreres et al.: only one deoxyhexosyl unit was attached to the C7-OH group, and all other acylated sugars were attached through the C3-OH.

On the basis of these, structures of the acylated quercetin- and kaempferol-*O*-glycosides could be proposed. The suggested structures of the compounds, together with the interpretation of the product ions that were considered characteristic or diagnostic, are presented in Table 2. Flavonol-*O*-acyl-glycosides were assigned to four major classes: (a) flavonol-*O*-coumaroyl and -caffeoyl-glycosides (compounds **50**, **52**, **53**, **69**, **82**, **84**, **86**, **87**, **89**, **90**, **92**); (b) flavonol-*O*-coumaroyl- and -feruloyl-glycosides (compounds **61**, **64**, **68**, **70**, **74**, **79**, **85**, **93**); (c) flavonol-*O*-feruloyl-sinapoyl-glycosides (compounds **57**, **67**); (d) flavonol-*O*-feruloyl-dihydrophaseoyl-glycosides (compounds **72**, **88**, **91**). It is worth noting that although Ferreres et al. reported flavonoid-acylglycosides of high molecular weight [23], the occurrence of flavonoids with molecular masses as high as 1508, 1880, or 1920 Da (compounds **67**, **75**, and **83**) is extremely rare. However, it should also be highlighted that this characterization is rather hypothetical; the unambiguous identification of the constituents was not possible. Compounds **46**, **75**, and **83** were also detected as doubly charged [M−2H]^2−^ ions at *m*/*z* 917, 939, and 959, respectively. However, except for compound **75**, other ions were not detected; therefore, we presumed these were also acylated flavonol-*O*-oligoglycosides. On the other hand, **75** displayed product ions at *m*/*z* 609 and 300, suggesting quercetin aglycone.

- Flavanone-, flavone-, and chalcone-glycosides

In addition to flavonol-*O*-glycosides, flavone-*O*- and -*C*-glycosides, as well as flavanone-*O*-glycosides and dihydrochalcone-*C*-glycosides, have also been detected in extracts of *L. tuberosus.* All flavone and flavanone-*O*-glycosides were characterized in the same way as those of the flavonol derivatives. Compound **19** was tentatively identified as tetrahydroxyflavanone-*O*-hexoside, based on its MS/MS fragmentation (*m*/*z* 449 → 287, 269, 259, 215) [29,30]. The compound **66** with its [M−H]^−^ ion at *m*/*z* 447 suffered a neutral loss of a hexose unit (*m*/*z* 447 → 285), while further fragmentation indicated the cleavage of a methoxy group (*m*/*z* 285 → 270); therefore, **66** was identified as dihydroxy-methoxyflavanone-*O*-hexoside [30,31]. Compound **49** showed that fragment ions at *m*/*z* 223 and 205 are typical for sinapic acid, while fragment ions at *m*/*z* 269 corresponded to the deprotonated apigenin aglycone. The neutral loss of 338 Da (*m*/*z* 607 → 269) indicated the fragmentation of a sinapoyl-pentose moiety; therefore, **49** was characterized as apigenin-7-*O*-sinapoyl-pentoside [22].

For the identification of *C*-glycosides, the neutral losses of 120 and 90 Da for *C*-hexoses and 90 and 60 Da for *C*-pentoses were characteristic [24,32]. According to the literature of other *Lathyrus* species, compound **39** at *m*/*z* 579 corresponded to luteolin-*C*-hexoside-*C*-pentoside [22,33]. Compound **12** exhibited the [M−H]^−^ ion at *m*/*z* 593 with its fragment ions at *m*/*z* 473, 383, 353, representing two neutral losses of 120 Da (*m*/*z* 593, 473, 353). Therefore, it was identified as apigenin-6-*C*-hexosyl-8-*C*-hexoside, previously reported in *Lathyrus* species [21]. Furthermore, dihydrochalcone-*C*-glycosides (compounds **24**, **30**, **32**, **58**, **73**, **76**, **78**, and **80**) were also tentatively characterized, comparing their MS spectra with the literature [34]. In addition to the common product ions generated by the characteristic neutral losses of *C*-glycosides (120, 90, and 60 Da), compounds **32** and **80** also produced unusual fragment ions [M−H−150]^−^ at *m*/*z* 417 and [M−H−162]^−^ at *m*/*z* 375, respectively [34,35]. Consequently, compounds **32**, **30**, and **80** were characterized as isomers of tetrahydroxy-dihydrochalcone-*C*-hexosyl-*C*-pentoside and tetrahydroxy-dihydrochalcone-di-C-pentoside, respectively. Based on the similarity of their mass spectra, a trihydroxy-dihydrochalcone-di-*C*-hexoside, and a trihydroxy-dihydrochalcone-di-*C*-pentoside structure were proposed for compounds **58** and **73**, respectively [34,35].

#### 2.1.2. Hydroxycinnamic Acid Derivatives

Characteristic fragment ions of hydroxycinnamic acid derivatives were used for their tentative characterization and already detailed in the section on the interpretation of the mass spectra of flavonol-*O*-acylglycosides. Pairs of product ions at *m*/*z* 179–161, 163–145, 223–205, and 193–175 were typical of caffeic acid, coumaric acid, sinapic acid, and ferulic acid, respectively. According to collision-induced dissociation (CID) of flavonoid glycosides, neutral losses of 162 and 132 Da pointed to cleavage of a hexose and a pentose moiety, respectively. Therefore, compound **1** with its [M−H]^−^ ion at *m*/*z* 341 was characterized as caffeoyl-*O*-hexoside [26]. Compound **11** exhibited its pseudomolecular ion at *m*/*z* 457, while successive cleavages of a pentose and a hexose moiety yielded the fragment ion [M−H−162−132]^−^ at *m*/*z* 163; therefore, compound **11** was described as *p*-coumaric acid-*O*-pentosyl-hexoside [36]. Similarly, compound **16** at *m*/*z* 385 was identified as sinapic acid-*O*-hexoside [37], while compounds **14**, **18**, and **22** at *m*/*z* 519, 387, and 681 were characterized as dihydrosinapic acid-*O*-pentosyl-hexoside, dihydrosinapic acid-*O*-hexoside, and dihydrosinapic acid-*O*-pentosyl-hexosyl-hexoside, respectively [38].

Compound **7** was identified as 5-*O*-caffeoyl-quinic acid based on fragment ion at *m*/*z* 191 [39]. Likewise, compound **45** was proposed as a 5-*O*-caffeoyl-quinic acid derivative. Compounds **51**, **54**, and **65** were characterized as dicaffeoylquinic acid isomers, which could be distinguished based on the relative intensities of their fragment ions [39]. For 3,4-*O*-dicaffeoylquinic acid (**51**), the fragment ion at *m*/*z* 173 is the base peak, while the intensity of the secondary fragment ions at *m*/*z* 191 and 179 is relatively high. The domination of the fragment ion at *m*/*z* 191 indicated 3,5-*O*-dicaffeoylquinic acid as compound **54**, while the base peak at *m*/*z* 173 with relatively low intensity secondary peaks referred to 4,5-*O*-dicaffeoylquinic acid (**65**) [39]. The presence of the latter two isomers was previously reported for *L. sativus* [22].

In the case of compound **3** at *m*/*z* 365, the loss of 162 Da indicated a caffeoyl moiety, while the fragment ions at *m*/*z* 203 and 159 referred to a tryptophan moiety, according to Llorent-Martínez et al. Therefore, this compound was tentatively identified as *N*-caffeoyltryptophan [40]. Compound **23** with the [M−H]^−^ ion at *m*/*z* 381, suffered the loss of a pentose (*m*/*z* 381 249), while the further loss of 88 Da (*m*/*z* 249 161) and the fragment ions at *m*/*z* 179 and 161 referred to a caffeoylputrescine derivative [41], therefore, **23** was characterized as *N*-caffeoylputrescine-*O*-pentoside. According to Gampe et al., non-protein amino acids (e.g., homoproline and homopipecolic acid) are widely distributed in species belonging to the Fabaceae family [42]. The appearance of the neurotoxin β-*N*-oxalyl-l-α,β-diaminopropionic acid (β-ODAP) in the seeds of *L. sativus* is also universally known [43]. However, hydroxycinnamoyl esters of amino acids, such as *N*-*trans*-caffeoyltyramine isolated from *Celtis occidentalis* L. [44], have not been reported in the genus *Lathyrus*.

Compound **60** presented a pseudomolecular ion at *m*/*z* 503 and fragment ions at *m*/*z* 371 [M−H−132]^−^ and 209 [M−H−132−162]^−^. The neutral losses corresponded to a pentose and a caffeoyl moiety, while the fragment ions at *m*/*z* 209 and 161 are characteristic of a tetrahydroxyhexanedioic acid (presumably galactaric acid) and a caffeoyl moiety, respectively. Therefore, compound **60** was identified as caffeoyl-tetrahydroxyhexanedioic acid-*O*-pentoside [45]. Fragment ions of compounds **8**, **15**, **28**, and **33**, at *m*/*z* 177 and 159 (the latter produced by the cleavage of a H_2_O molecule) indicated the presence of a dimethyl-dihydroxybutanedioic acid (probably dimethyltartaric acid). In the case of compounds **8**, **28**, and **33**, the neutral loss of 146 Da and the fragment ion at *m*/*z* 163 pointed to a coumaroyl moiety, while for compound **15**, the loss of 180 Da and the fragment ion at *m*/*z* 179 indicated a caffeoyl moiety. Therefore, **28** and **33** were identified as coumaroyl-dimethyl-dihydroxybutanedioic acid isomers, and compound **15** as a caffeoyl derivative [22]. Compound **8**, showing an additional neutral loss of 162 Da, was characterized as coumaroyl-dimethyl-dihydroxybutanedioic acid-*O*-hexoside.

#### 2.1.3. Hydroxybenzoic Acid Derivatives

The fragmentation patterns of compounds **4** and **6** indicated the presence of dihydroxybenzoic acid and hydroxybenzoic acid moieties, respectively. Compound **4** at *m*/*z* 417 presented fragment ions [M−H−132−44]^−^ at *m*/*z* 241, [dihydroxybenzoic acid−H]^−^ at *m*/*z* 153, and [M−H−132−133]^−•^ at *m*/*z* 152, therefore, it was characterized as dihydroxybenzoic acid-di-*O*-pentoside [34]. In the case of compound **6**, the neutral loss of 162 Da (a hexose moiety) yielded the [hydroxybenzoic acid−H]^−^ ion at *m*/*z* 137; therefore, **6** at *m*/*z* 299 was identified as *p*-hydroxybenzoic acid-*O*-hexoside [46]. Based on the literature, compound **10** was tentatively identified as hydroxybenzyl-*O*-malic acid (eucomic acid), already reported in legumes [47].

#### 2.1.4. Lignan Derivatives

Compounds **13**, **21**, and **25** at *m*/*z* 551 were characterized as methoxylariciresinol-*O*-hexoside isomers [48], while compounds **2** and **9** with [M−H]^−^ at *m*/*z* 713 consisting of an additional hexose moiety (*m*/*z* 713 → 551) were identified as methoxylariciresinol-di-*O*-hexoside isomers [49]. Compound **77** exhibited the molecular ion at *m*/*z* 519 with a neutral loss of 162 Da that yielded an aglycone ion at *m*/*z* 357, while the other fragment ions showed similarity to epipinoresinol or pinoresinol [50,51]. In the case of compounds **38** and **55**, the aglycone fragment ions [M−H−162]^−^ and [M−H−132]^−^ at *m*/*z* 373 contain an extra hydroxyl group, and their [M−H]^−^ ions can be detected at *m*/*z* 535 and 505, respectively. Thus, they were characterized as hydroxypinoresinol-*O*-hexoside (**38**) and hydroxypinoresinol-*O*-pentoside (**55**) [50,51].

#### 2.1.5. Monoterpene Derivatives

Compound **48** presented a pseudomolecular ion at *m*/*z* 523 and fragment ions at *m*/*z* 361 (indicating the loss of a caffeoyl moiety) and 161 (characteristic of caffeoyl moiety). Based on the literature, these characteristics suggested the structure of verminoside consisting of the iridoid glucoside catalpol linked to a caffeoyl moiety [52]. Constituent **40** that exhibited the [M−H]^−^ ion at *m*/*z* 371 was identified as deacetylasperuloside [53].

**Table 1:** LC-MS/MS data and tentative characterization of compounds from the herb of *Lathyrus tuberosus*.

No. ^a^	Tentative Characterization	Presence of Compounds		t_R_ (min) ^a^	[M−H]^−^ (*m*/*z*)	Fragment Ions (*m*/*z*)	Ref.
		LtE ^b^	LtW ^b^				
1	caffeic acid-*O*-hexoside	+	-	0.80	341 377 [M+Cl]^−^	179, 161, 149, 119	[26]
2	methoxylariciresinol-di-*O*-hexoside isomer	+	+	0.94	713	551, 173, 135	[49]
3	*N*-caffeoyltryptophan	+	+	0.97	365	203, 159	[40]
4	dihydroxybenzoic acid-di-*O*-pentoside	+	+	1.00	417	241, 152	[34]
5	unknown	+	+	1.21	813	353	-
6	*p*-hydroxybenzoic acid-*O*-hexoside	+	+	1.27	299	228, 137	[46]
7	5-*O*-caffeoylquinic acid	+	+	1.27	353 707 [2M−H]^−^	191	[39]
8	coumaroyl-dimethyl-dihydroxybutanedioic acid-*O*-hexoside	+	+	1.36	485	323, 163, 159	-
9	methoxylariciresinol-di-*O*-hexoside isomer	+	+	1.42	713	551	[49]
10	hydroxybenzyl-*O*-malic acid (eucomic acid)	+	+	1.42	239	179, 149, 133, 107	[47]
11	*p*-coumaric acid-*O*-pentosyl-hexoside	+	+	1.45	457	163	[36]
12	apigenin-6-*C*-hexosyl-8-*C*-hexoside	+	+	1.62	593	473, 383, 353	[21]
13	methoxylariciresinol-*O*-hexoside isomer	+	+	1.65	551	389, 371, 341, 285, 193, 165, 149	[48]
14	dihydrosinapic acid-*O*-pentosyl-hexoside	+	+	1.79	519	387, 233, 207, 189, 161	[38]
15	caffeoyl-dimethyl-dihydroxybutanedioic acid	+	+	2.04	339	179, 159	[22]
16	sinapic acid-*O*-hexoside	+	+	2.05	385	205, 153, 119	[37]
17	quercetin-3-*O*-hexosyl-7-*O*-deoxyhexoside isomer	+	-	2.08	609	463, 447, 446, 301, 300, 299	[21,26]
18	dihydrosinapic acid-*O*-hexoside	+	+	2.10	387	207, 189, 153, 119	[38]
19	tetrahydroxyflavanone-*O*-hexoside	+	+	2.10	449	287, 269, 259, 215, 149, 125	[29,30]
20	unknown	+	+	2.11	813	-	-
21	methoxylariciresinol-*O*-hexoside isomer	+	+	2.28	551	389, 341, 285, 193, 165, 149	[48]
22	dihydrosinapic acid-*O*-pentosyl-hexosyl-hexoside	+	+	2.34	681	519, 387, 339, 309, 233, 207, 203, 189, 179, 161, 149, 123, 119, 113	[38]
23	*N*-caffeoylputrescine-*O*-pentoside	+	+	2.34	381	249, 179, 161, 113, 101	[41]
24	tetrahydroxy-dihydrochalcone-*C*-glycoside derivative	+	+	2.38	517	417, 399, 163, 152	[34]
25	methoxylariciresinol-*O*-hexoside isomer	+	+	2.48	551	193, 165, 151	[48]
26	quercetin-*O*-deoxyhexosyl-hexosyl-pentoside	+	+	2.67	741	300	[23]
27	quercetin-3-*O*-hexosyl-7-*O*-deoxyhexoside isomer	+	+	2.96	609	463, 301, 300, 299	[21,26]
28	coumaroyl-dimethyl-dihydroxybutanedioic acid isomer	+	+	3.15	323	177, 163, 159, 141, 131, 119	-
29	quercetin-3-*O*-deoxyhexosyl-7-*O*-deoxyhexoside	+	+	3.22	593	447, 446, 301, 300, 299	[28]
30	tetrahydroxy-dihydrochalcone-di-*C*-pentoside isomer	+	+	3.25	537	417, 399, 179, 152, 137	[34]
31	isorhamnetin-*O*-hexosyl-deoxyhexoside or verbascoside (acteoside)	+	-	3.45	623	477, 461, 315	[21,22]
32	tetrahydroxy-dihydrochalcone-*C*-hexosyl-*C*-pentoside	+	+	3.57	567	549, 417, 399, 357, 209, 195, 167, 165, 153, 152, 137, 119	[34,35]
33	coumaroyl-dimethyl-dihydroxybutanedioic acid isomer	+	+	3.70	323	177, 159, 141, 131, 119	-
34	quercetin-*O*-hexosyl-hexosyl-hexosyl-deoxyhexoside isomer	+	-	3.70	933	-	[21]
35	quercetin-3-*O*-deoxyhexosyl-hexoside	+	-	3.75	609	301, 300, 179, 151	[21,26]
36	kaempferol-3-*O*-pentosyl-7-*O*-glucuronide	+	+	3.99	593	461, 460, 284, 283, 257, 179, 163	[23]
37	quercetin-3-*O*-hexoside isomer	+	+	4.11	463	300, 271, 151	[22,26]
38	hydroxypinoresinol-*O*-hexoside	+	-	4.20	535	373, 233, 209, 163, 119	[50,51]
39	luteolin-*C*-hexosyl-*C*-pentoside	+	+	4.24	579	339, 327	[22,33]
40	deacetylasperuloside	+	-	4.28	371	233, 209, 165, 125	[53]
41	quercetin-3-*O*-hexosyl-7-*O*-hexosyl-hexosyl-deoxyhexoside isomer	+	+	4.28	933	609, 463, 301, 300, 299, 271, 179	[21]
42	quercetin-3-*O*-hexoside isomer	+	+	4.36	463	300, 271, 179, 151	[22,26]
43	unknown	+	+	4.36	501	-	-
44	kaempferol-3-*O*-deoxyhexosyl-7-*O*-deoxyhexoside	+	+	4.75	577	431, 430, 285, 284, 283	[25]
45	5-*O*-caffeoylquinic acid derivative	+	+	4.80	503	191, 161, 149	-
46	acylated flavonoid-*O*-oligoglycoside	+	+	5.03	1835 917 [M−2H]^2−^	-	[23]
47	unknown	+	+	5.11	951	-	-
48	verminoside	+	-	5.23	523	361, 161, 101	[52]
49	apigenin-7-*O*-sinapoyl-pentoside	+	-	5.28	607	269, 223, 205	[22]
50	quercetin-*O*-deoxyhexosyl-hexosyl-coumaroyl-hexosyl-caffeoyldihexoside	+	+	5.28	1403 701 [M−2H]^2−^	1247, 917, 609, 301, 300, 211, 161, 145	[23]
51	3,4-di-*O*-caffeoylquinic acid	+	+	5.28	515	353, 335, 191, 179, 173, 161, 135	[39]
52	quercetin-*O*-coumaroyldihexosyl-coumaroylhexosyl-deoxyhexosyl-hexoside isomer	+	+	5.45	1387 693 [M−2H]^2−^	1241, 1175, 1079, 933, 771, 609, 301, 300, 211, 163, 145	[23]
53	quercetin-*O*-coumaroyldihexosyl-coumaroylhexosyl-deoxyhexosyl-hexoside isomer	+	+	5.63	1387 693 [M−2H]^2−^	1233, 1175, 1079, 933, 609, 463, 462, 301, 300, 299, 211, 163, 145	[23]
54	3,5-di-*O*-caffeoylquinic acid	+	+	5.80	515 537 [M+Na−2H]^−^	353, 191, 179, 173, 135	[22,39]
55	hydroxypinoresinol-*O*-pentoside	+	+	6.00	505 541 [M+Cl]^−^	373, 161, 113	[50,51]
56	quercetin-3-*O*-deoxyhexosyl-hexosyl-7-*O*-deoxyhexosyl-hexoside	+	+	6.28	917	771, 609, 608, 463, 445, 301, 300, 299	[21]
57	kaempferol-*O*-sinapoylhexosyl-feruloylpentosyl-dicaffeoyl-hexoside	+	+	6.30	1447 723 [M−2H]^2−^	1241, 1079, 771, 593, 285, 284, 283, 223, 205, 193, 179, 175, 161	[23]
58	trihydroxy-dihydrochalcone-di-*C*-hexoside	+	+	6.33	581	399, 381, 167, 152	[34]
59	unknown	+	+	6.40	501	-	-
60	caffeoyl-tetrahydroxyhexanedioic acid-*O*-pentoside	+	+	6.70	503	371, 209, 161, 113	[45]
61	quercetin-*O*-coumaroyldihexosyl-feruloylpentosyl-deoxyhexosyl-hexoside isomer	+	+	6.75	1387 693 [M−2H]^2−^	1241, 1225, 933, 609, 301, 300, 299, 211, 193, 175, 163, 145	[23]
62	unknown	+	+	6.82	459	165	-
63	quercetin-3-*O*-deoxyhexosyl-7-*O*-hexosyl-hexosyl-glucuronide	+	+	7.01	947	771, 625, 463, 447, 301, 300, 299, 271, 255	[21]
64	quercetin-*O*-di(feruloylhexosyl)-hexosyl-deoxyhexosyl-hexoside	+	+	7.31	1447 723 [M−2H]^2−^	1271, 789, 609, 301, 300, 299, 193, 175	[23]
65	4,5-di-*O*-caffeoylquinic acid	+	+	7.33	515	353, 191, 179, 173, 135	[22,39]
66	dihydroxy-methoxyflavanone-*O*-hexoside	+	+	7.45	447	285, 270, 145	[30,31]
67	quercetin-*O*-sinapoyldihexosyl-feruloyldeoxyhexosyl-deoxyhexosyl-hexoside formiate adduct	+	+	7.52	1507 753 [M−2H]^2−^	1255, 931, 609, 301, 300, 299, 205, 175	[23]
68	kaempferol-*O*-coumaroylhexosyl-feruloylhexosyl-pentosyl-deoxyhexosyl-hexoside isomer	+	+	7.57	1371 685 [M−2H]^2−^	1225, 1093, 931, 593, 285, 284, 227, 193, 175, 145	[23,24]
69	kaempferol-*O*-caffeoylhexosyl-caffeoyldihexosyl-deoxyhexosyl-hexoside	+	+	7.59	1403 701 [M−2H]^2−^	917, 593, 285, 241, 227, 179	[23]
70	quercetin-*O*-coumaroyldihexosyl-feruloylpentosyl-deoxyhexosyl-hexoside isomer	+	+	7.63	1387 693 [M−2H]^2−^	1241, 1225, 1173, 933, 917, 609, 301, 300, 193, 175, 163, 145	[23]
71	quercetin-3-*O*-acetylhexoside		+	7.85	505	301, 300, 151	[21]
72	quercetin-3-*O*-dihydrophaseoyl-deoxyhexosyl-feruloylhexosyl-7-*O*-deoxyhexoside	+	+	8.05	1195	1049, 931, 785, 609, 301, 300, 299, 175	[23]
73	trihydroxy-dihydrochalcone-di-*C*-pentoside	+	+	8.20	521	399, 327, 267, 207, 153, 152, 109	[34]
74	kaempferol-*O*-coumaroylhexosyl-feruloylhexosyl-pentosyl-deoxyhexosyl-hexoside isomer	+	+	8.33	1371 685 [M−2H]^2−^	1225, 1093, 593, 285, 284, 175, 163, 145	[23,24]
75	acylated quercetin-*O*-oligoglycoside	+	+	8.35	1879 939 [M−2H]^2−^	609, 300	-
76	trihydroxy-dihydrochalcone-*C*-glycoside derivative	+	+	8.42	501	399, 327, 267, 207, 163, 152	[34]
77	epipinoresinol/pinoresinol-*O*-hexoside	+	+	8.50	519	357, 343, 341, 161	[50,51]
78	trihydroxy-dihydrochalcone-*C*-glycoside derivative	+	+	8.75	501	437, 417, 399, 152	[34]
79	quercetin-*O*-feruloylhexosyl-coumaroylpentosyl-acetylhexosyl-deoxyhexosyl-hexoside	+	+	8.87	1429 714 [M−2H]^2−^	1091, 959, 813, 651, 609, 608, 301, 300, 299, 211, 193, 175, 163, 159, 145	[23,24]
80	tetrahydroxy-dihydrochalcone-di-*C*-pentoside isomer	+	+	8.97	537	417, 399, 267, 152, 137, 108	[34,35]
81	kaempferol-3-*O*-deoxyhexoside	-	+	9.00	431	285, 255	[21,25,26]
82	quercetin-*O*-di(coumaroylhexosyl)-acetylhexosyl-deoxyhexosyl-hexoside	+	+	9.00	1429 714 [M−2H]^2−^	1267, 1217, 651, 609, 463, 301, 300, 299, 211, 163, 145	[23,24]
83	acylated flavonoid-*O*-oligoglycoside	+	+	9.43	1919 959 [M−2H]^2−^	-	-
84	kaempferol-*O*-di(coumaroylhexosyl)-acetylhexosyl-deoxyhexosyl-hexoside	+	+	9.50	1413 706 [M−2H]^2−^	593, 285, 284, 229, 211, 163, 145	[23]
85	quercetin-*O*-coumaroyldihexosyl-feruloylpentosyl-deoxyhexosyl-hexoside isomer	+	+	9.55	1387 693 [M−2H]^2−^	609, 301, 300, 211, 193, 175, 163, 145	[23]
86	quercetin-*O*-(coumaroyl-caffeoyl)-deoxyhexosyl-hexosyl-acetylhexosyl-glucuronyl-pentoside isomer	+	+	9.70	1429 714 [M−2H]^2−^	1283, 959, 651, 609, 608, 433, 301, 300, 299, 211, 179, 145	[23]
87	kaempferol-*O*-di(coumaroylhexosyl)-acetylhexosyl-deoxyhexosyl-hexoside isomer	+	+	9.75	1413 706 [M−2H]^2−^	635, 593, 285, 284, 283, 229, 211, 163, 145	[23]
88	kaempferol-3-*O*-dihydrophaseoyl-hexosyl-diferuloylhexosyl-7-*O*-deoxyhexoside	+	+	9.85	1371 685 [M−2H]^2−^	1195, 593, 285, 284, 283	[23]
89	quercetin-*O*-(coumaroyl-caffeoyl)-deoxyhexosyl-hexosyl-acetylhexosyl-glucuronyl-pentoside isomer	+	+	10.13	1429 714 [M−2H]^2−^	1267, 1225, 1093, 975, 651, 609, 608, 433, 301, 300, 299, 211, 179, 145	[23]
90	kaempferol-*O*-di(coumaroylhexosyl)-acetylhexosyl-deoxyhexosyl-hexoside isomer	+	+	10.31	1413 706 [M−2H]^2−^	1267, 635, 593, 285, 284, 283, 211, 163, 145	[23]
91	quercetin-3-*O*-dihydrophaseoyl-deoxyhexosyl-diferuloylhexosyl-7-*O*-deoxyhexoside	+	+	10.50	1371 685 [M−2H]^2−^	1195, 1107, 609, 301, 300, 299, 195, 193, 175, 145	[23]
92	quercetin-*O*-di(coumaroylhexosyl)-acetylhexosyl-deoxyhexosyl-hexoside isomer	+	+	10.66	1429 714 [M−2H]^2−^	1267, 1121, 651, 609, 608, 301, 300, 299, 211, 145	[23]
93	quercetin-coumaroylpentosyl-(feruloyl-caffeoyl)-deoxyhexosyl-deoxyhexosyl-hexoside	+	+	11.20	1371 685 [M−2H]^2−^	1225, 1093, 609, 301, 300, 195, 193, 179, 175, 145	[23]

^a^ Compound numbers and retention times (t_R_) refer to UV chromatograms shown in Figures S1 and S2; ^b^ Abbreviations: LtE: *Lathyrus tuberosus* 50% (*v*/*v*) ethanolic extract; LtW: *Lathyrus tuberosus* aqueous extract; +: present in the extract; -: not present in the extract.

**Table 2:** LC-MS/MS data of flavonol-*O*-glycosides and acylated flavonol-*O*-glycosides from the herb of *Lathyrus tuberosus.*

**Flavonol-*O*-glycosides**					
**No. ^a^**	**Tentative Characterization**	**t_R_ (min) ^a^**	**[M−H]^−^** **(*m*/*z*)**	**Fragment Ions** **(*m*/*z*)**	**Ref.**
17	quercetin-3-*O*-hexosyl-7-*O*-deoxyhexoside isomer	2.08	609	**463** [M−H−146]^−^, **447** [M−H−162]^−^, **446** [M−H−^•^163]^−•^, **301** [M−H−146−162]^−^ = Y_0_^−^, **300** [M−H−146−^•^163]^−•^ = [Y_0_−H]^−•^, **299** [M−H−^•^147−^•^163]^−^ = [Y_0_−2H]^−^	[21,26]
27	quercetin-3-*O*-hexosyl-7-*O*-deoxyhexoside isomer	2.96	609	**463** [M−H−146]^−^, **301** [M−H−146−162]^−^ = Y_0_^−^, **300** [M−H−146−^•^163]^−•^ = [Y_0_−H]^−•^, **299** [M−H−^•^147−^•^163]^−^ = [Y_0_−2H]^−^	[21,26]
29	quercetin-3-*O*-deoxyhexosyl-7-*O*-deoxyhexoside	3.22	593	**447** [M−H−146]^−^, **446** [M−H−^•^147] ^−•^, **301** [M−H−146−146]^−^ = Y_0_^−^, **300** [M−H−146−^•^147]^−•^ = [Y_0_−H]^−•^, **299** [M−H−^•^147−^•^147]^−^ = [Y_0_−2H]^−^	[28]
31	isorhamnetin-*O*-hexosyl-deoxyhexoside	3.45	623	**477** [M−H−146]^−^, **461** [M−H−162]^−^, **315** [M−H−146−162]^−^ = Y_0_^−^	[21,22]
35	quercetin-3-*O*-deoxyhexosyl-hexoside	3.75	609	**301** [M−H−146−162]^−^ = Y_0_^−^, **300** [M−H−^•^147−162]^−•^ or [M−H−146−^•^163]^−•^ = [Y_0_−H]^−^	[21,26]
36	kaempferol-3-*O*-pentosyl-7-*O*-glucuronide	3.99	593	**461** [M−H−132]^−^, **460** [M−H−^•^133]^−•^, **284** [M−H−^•^133−176]^−•^ = [Y_0_−H]^−•^, **283** [M−H−^•^133−^•^177]^−^ = [Y_0_−2H]^−^, **257** [Y_0_−CO]^−^	[23]
37	quercetin-3-*O*-hexoside isomer	4.11	463	**300** [M−H−^•^163]^−•^ = [Y_0_−H]^−•^, **271** [Y_0_−H−CO−H]^−^	[22,26]
41	quercetin-3-*O*-hexosyl-7-*O*-hexosyl-hexosyl-deoxyhexoside isomer	4.28	933	**609** [M−H−162−162]^−^, **463** [M−H−162−162−146]^−^, **301** [M−H−162−162−146−162]^−^ = Y_0_^−^, **300** [Y_0_−H]^−•^, **299** [Y_0_−2H]^−^, **271** [Y_0_−H−CO−H]^−^	[21]
42	quercetin-3-*O*-hexoside isomer	4.36	463	**300** [M−H−^•^163]^−•^ = [Y_0_−H]^−•^, **271** [Y_0_−H−CO−H]^−^	[22,26]
44	kaempferol-3-*O*-deoxyhexosyl-7-*O*-deoxyhexoside	4.75	577	**431** [M−H−146]^−^, **430** [M−H−^•^147]^−•^, **285** [M−H−146−146]^−^ = Y_0_^−^, **284** [M−H−146−^•^147]^−•^ = [Y_0_−H]^−•^, **283** [M−H−^•^147−^•^147]^−^ = [Y_0_−2H]^−^	[25]
56	quercetin-3-*O*-deoxyhexosyl-hexosyl-7-*O*-deoxyhexosyl-hexoside	6.28	917	**771** [M−H−146]^−^, **609** [M−H−146−162]^−^, **608** [M−H−146−^•^163]^−•^, **463** [M−H−146−162−146]^−^, **301** [M−H−146−162−146−162]^−^ = Y_0_^−^, **300** [M−H−146−162−146−^•^163]^−•^ = [Y_0_−H]^−•^, **299** [Y_0_−2H]^−^	[21]
63	quercetin-3-*O*-deoxyhexosyl-7-*O*-hexosyl-hexosyl-glucuronide	7.01	947	**771** [M−H−176]^−^, **625** [M−H−176−146]^−^, **463** [M−H−176−146−162]^−^, **447** [M−H−176−162−162]^−^, **301** [M−H−176−162−162−146]^−^ = Y_0_^−^, **300** [Y_0_−H]^−•^, **299** [Y_0_−2H]^−^, **271** [Y_0_−H−CO−H]^−^, **255** [Y_0_−H−CO_2_−H]^−^	[21]
71	quercetin-3-*O*-acetylhexoside	7.85	505	**301** [M−H−42−162]^−^ = Y_0_^−^, **300** [M−H−42−^•^163]^−•^ = [Y_0_−H]^−•^	[21]
81	kaempferol-3-*O*-deoxyhexoside	9.00	431	**285** [M−H−146]^−^ = Y_0_^−^, **255** [Y_0_−H−CO−H]^−^	[21,26,27]
**Flavonol-*O*-coumaroyl- and -caffeoyl-glycosides**					
**No. ^a^**	**Tentative Characterization**	**t_R_ (min) ^a^**	**[M−H]^−^** **(*m*/*z*)**	**Fragment ions** **(*m*/*z*)**	**Ref.**
50	quercetin-*O*-coumaroylhexosyl-caffeoyldihexosyl-deoxyhexosyl-hexoside	5.28	1403 701 [M−2H]^2−^	**917** [M−H−162−162−162]^−^, **609** [M−H−162−162−162−146−162]^−^, **301** [M−H−162−162−162−146−162−146−162]^−^ = Y_0_^−^, **300** [Y_0_−H]^−•^, **161** [caffeoyl−H]^−^, **145** [coumaroyl−H]^−^	[23]
52	quercetin-*O*-coumaroyldihexosyl-coumaroylhexosyl-deoxyhexosyl-hexoside isomer	5.45	1387 693 [M−2H]^2−^	**1241** [M−H−146]^−^, **1079** [M−H−146−162]^−^, **933** [M−H−146−162−146]^−^, **771** [M−H−146−162−146−162]^−^, **609** [M−H−146−162−146−162−162]^−^, **301** [M−H−146−162−146−162−162−146−162]^−^ = Y_0_^−^, **300** [Y_0_−H] ^•^, **163** [coumaric acid−H]^−^, **145** [coumaroyl−H]^−^	[23]
53	quercetin-*O*-coumaroyldihexosyl-coumaroylhexosyl-deoxyhexosyl-hexoside isomer	5.63	1387 693 [M−2H]^2−^	**1079** [M−H−146−162]^−^, **933** [M−H−146−162−146]^−^, **609** [M−H−146−162−146−162−162]^−^, **463** [M−H−146−162−146−162−162−146]^−^, **462** [M−H−146−162−146−162−162−^•^147]^−•^, **301** [M−H−146−162−146−162−162−146−162]^−^ = Y_0_^−^, **300** [Y_0_−H]^−•^, **299** [Y_0_−2H]^−^, **163** [coumaric acid−H]^−^, **145** [coumaroyl−H]^−^	[23]
69	kaempferol-*O*-caffeoylhexosyl-caffeoyldihexosyl-deoxyhexosyl-hexoside	7.59	1403 701 [M−2H]^2−^	**917** [M−H−162−162−162]^−^, **593** [M−H−162−162−162−162−162]^−^, **285** [M−H−162−162−162−162−162−162−146]^−^ = Y_0_^−^, **241** [Y_0_−H−CO_2_]^−^, **227** [Y_0_−H−2CO−H]^−^, **179** [caffeic acid−H]^−^	[23]
82	quercetin-*O*-di(coumaroylhexosyl)-acetylhexosyl-deoxyhexosyl-hexoside isomer	9.00	1429 714 [M−2H]^2−^	**1267** [M−H−162]^−^, 1217, **651** [M−H−162−146−146−162−162]^−^, **609** [M−H−162−146−146−162−162−42]^−^, **463** [M−H−162−146−146−162−162−42−146]^−^, **301** [M−H−162−146−146−162−162−42−146−162]^−^ = Y_0_^−^, **300** [Y_0_−H]^−•^, **299** [Y_0_−2H]^−^, **163** [coumaric acid−H]^−^, **145** [coumaroyl−H]^−^	[23,24]
84	kaempferol-*O*-di(coumaroylhexosyl)-acetylhexosyl-deoxyhexosyl-hexoside isomer	9.50	1413 706 [M−2H]^2−^	**593** [M−H−162−146−146−162−162−42]^−^, **285** [M−H−162−146−146−162−162−42−162−146]^−^ = Y_0_^−^, **284** [Y_0_−H]^−•^, **163** [coumaric acid−H]^−^, **145** [coumaroyl−H]^−^	[23]
86	quercetin-*O*-(coumaroyl-caffeoyl)-deoxyhexosyl-hexosyl-acetylhexosyl-glucuronyl-pentoside isomer	9.70	1429 714 [M−2H]^2−^	**1283** [M−H−146]^−^, **959** [M−H−146−162−162]^−^, **651** [M−H−146−162−162−146−162]^−^, **609** [M−H−146−162−162−146−162−42]^−^, **608** [M−H−146−162−162−146−^•^163−42]^−•^, **433** [M−H−146−162−162−146−162−42−176]^−^, **301** [M−H−146−162−162−146−162−42−176−132]^−^ = Y_0_^−^, **300** [Y_0_−H]^−•^, **299** [Y_0_−2H]^−^, **179** [caffeic acid−H]^−^, **145** [coumaroyl−H]^−^	[23]
87	kaempferol-*O*-di(coumaroylhexosyl)-acetylhexosyl-deoxyhexosyl-hexoside isomer	9.75	1413 706 [M−2H]^2−^	**635** [M−H−162−146−146−162−162]^−^, **593** [M−H−162−146−146−162−162−42]^−^, **285** [M−H−162−146−146−162−162−42−162−146]^−^ = Y_0_^−^, **284** [Y_0_−H]^−•^, **283** [Y_0_−2H]^−^, **163** [coumaric acid−H]^−^, **145** [coumaroyl−H]^−^	[23]
89	quercetin-*O*-(coumaroyl-caffeoyl)-deoxyhexosyl-hexosyl-acetylhexosyl-glucuronyl-pentoside isomer	10.13	1429 714 [M−2H]^2−^	**1267** [M−H−162]^−^, **1225** [M−H−162−42]^−^, **1093** [M−H−162−42−132]^−^, **975** [M−H−162−146−146]^−^, **651** [M−H−162−146−146−162−162]^−^, **609** [M−H−162−146−146−162−162−42]^−^ or [M−H−162−42−132−176−162−146]^−^, **433** [M−H−162−146−146−162−162−42−176]^−^, **301** [M−H−162−146−146−162−162−42−176−132]^−^ = Y_0_^−^, **300** [Y_0_−H]^−•^, **299** [Y_0_−2H]^−^, **179** [caffeic acid−H]^−^, **145** [coumaroyl−H]^−^	[23]
90	kaempferol-*O*-di(coumaroylhexosyl)-acetylhexosyl-deoxyhexosyl-hexoside isomer	10.31	1413 706 [M−2H]^2−^	**1267** [M−H−146]^−^, **635** [M−H−146−146−162−162−162]^−^, **593** [M−H−146−146−162−162−162−42]^−^, **285** [M−H−146−146−162−162−162−42−162−146]^−^ = Y_0_^−^, **284** [Y_0_−H]^−•^, **283** [Y_0_−2H]^−^, **163** [coumaric acid−H]^−^, **145** [coumaroyl−H]^−^	[23]
92	quercetin-*O*-di(coumaroylhexosyl)-acetylhexosyl-deoxyhexosyl-hexoside isomer	10.66	1429 714 [M−2H]^2−^	**1267** [M−H−162]^−^, **1121** [M−H−162−146]^−^, **651** [M−H−162−146−162−162−146]^−^, **609** [M−H−162−146−162−162−146−42]^−^, **608** [M−H−162−146−162−^•^163−146−42]^−•^, **301** [M−H−162−146−162−162−146−42−162−146]^−^ = Y_0_^−^, **300** [Y_0_−H]^−•^, **299** [Y_0_−2H]^−^, **145** [coumaroyl−H]^−^	[23]
**Flavonol-*O*-coumaroyl- and -feruloyl-glycosides**					
**No. ^a^**	**Tentative Characterization**	**t_R_ (min) ^a^**	**[M−H]^−^** **(*m*/*z*)**	**Fragment ions** **(*m*/*z*)**	**Ref.**
61	quercetin-*O*-coumaroyldihexosyl-feruloylpentosyl-deoxyhexosyl-hexoside isomer	6.75	1387 693 [M−2H]^2−^	**1241** [M−H−146]^−^, **1225** [M−H−162]^−^, **933** [M−H−146−162−146]^−^, **609** [M−H−146−162−146−162−162]^−^, **301** [M−H−146−162−146−162−162−146−162]^−^ = Y_0_^−^, **300** [Y_0_−H]^−•^, **299** [Y_0_−2H]^−^, **193** [ferulic acid−H]^−^, **175** [feruloyl−H]^−^, **163** [coumaric acid−H]^−^, **145** [coumaroyl−H]^−^	[23]
64	quercetin-*O*-di(feruloylhexosyl)-hexosyl-deoxyhexosyl-hexoside	7.31	1447 723 [M−2H]^2−^	**1271** [M−H−176]^−^, **609** [M−H−176−162−162−162−176]^−^, **301** [M−H−176−162−162−162−176−146−162]^−^ = Y_0_^−^, **300** [Y_0_−H]^−•^, **299** [Y_0_−2H]^−^, **193** [ferulic acid−H]^−^, **175** [feruloyl−H]^−^	[23]
68	kaempferol-*O*-coumaroylhexosyl-feruloylhexosyl-pentosyl-deoxyhexosyl-hexoside isomer	7.57	1371 685 [M−2H]^2−^	**1225** [M−H−146]^−^, **1093** [M−H−146−132]^−^, **931** [M−H−146−132−162]^−^, **593** [M−H−146−132−162−338]^−^, **285** [M−H−146−132−162−338−162−146]^−^ = Y_0_^−^, **284** [Y_0_−H]^−•^, **227** [Y_0_−H−2CO−H]^−^, **193** [ferulic acid−H]^−^, **175** [feruloyl−H]^−^, **145** [coumaroyl−H]^−^	[23,24]
70	quercetin-*O*-coumaroyldihexosyl-feruloylpentosyl-deoxyhexosyl-hexoside isomer	7.63	1387 693 [M−2H]^2−^	**1241** [M−H−146]^−^, **1225** [M−H−162]^−^, **933** [M−H−162−146−146]^−^ or [M−H−146−176−132]^−^, **917** [M−H−162−162−146]^−^ or [M−H−162−176−132]^−^, **609** [M−H−162−162−146−176−132]^−^, **301** [M−H−162−162−146−176−132−162−146]^−^ = Y_0_^−^, **300** [Y_0_−H]^−•^, **193** [ferulic acid−H]^−^, **175** [feruloyl−H]^−^, **163** [coumaric acid−H]^−^, **145** [coumaroyl−H]^−^	[23]
74	kaempferol-*O*-coumaroylhexosyl-feruloylhexosyl-pentosyl-deoxyhexosyl-hexoside isomer	8.33	1371 685 [M−2H]^2−^	**1225** [M−H−146]^−^, **1093** [M−H−146−132]^−^, **593** [M−H−146−132−162−162−176]^−^, **285** [M−H−146−132−162−162−176−162−146]^−^ = Y_0_^−^, **284** [Y_0_−H]^−•^, **175** [feruloyl−H]^−^, **163** [coumaric acid−H]^−^, **145** [coumaroyl−H]^−^	[23,24]
79	quercetin-*O*-feruloylhexosyl-coumaroylpentosyl-acetylhexosyl-deoxyhexosyl-hexoside	8.87	1429 714 [M−2H]^2−^	**1091** [M−H−338]^−^, **959** [M−H−338−132]^−^, **813** [M−H−338−132−146]^−^, **651** [M−H−338−132−146−162]^−^, **609** [M−H−338−132−146−162−42]^−^, **301** [M−H−338−132−146−162−42−162−146]^−^ = Y_0_^−^, **300** [Y_0_−H]^−•^, **299** [Y_0_−2H]^−^, **193** [ferulic acid−H]^−^, **175** [feruloyl−H]^−^, **163** [coumaric acid−H]^−^, **145** [coumaroyl−H]^−^	[23,24]
85	quercetin-*O*-coumaroyldihexosyl-feruloylpentosyl-deoxyhexosyl-hexoside isomer	9.55	1387 693 [M−2H]^2−^	**609** [M−H−162−162−146−176−132]^−^, **301** [M−H−162−162−146−176−132−162−146]^−^ = Y_0_^−^, **300** [Y_0_−H]^−•^, **193** [ferulic acid−H]^−^, **175** [feruloyl−H]^−^, **163** [coumaric acid−H]^−^, **145** [coumaroyl−H]^−^	[23]
93	quercetin-coumaroylpentosyl-(feruloyl-caffeoyl)-deoxyhexosyl-deoxyhexosyl-hexoside	11.20	1371 685 [M−2H]^2−^	**1225** [M−H−146]^−^, **1093** [M−H−146−132]^−^, **609** [M−H−146−132−162−176−146]^−^, **301** [M−H−146−132−162−176−146−162−146]^−^ = Y_0_^−^, **300** [Y_0_−H]^−•^, **193** [ferulic acid−H], **179** [caffeic acid−H]^−^, **175** [feruloyl−H]^−^, **145** [coumaroyl−H]^−^	[23]
**Flavonol-*O*-feruloyl-sinapoyl-glycosides**					
**No. ^a^**	**Tentative Characterization**	**t_R_ (min) ^a^**	**[M−H]^−^** **(*m*/*z*)**	**Fragment ions** **(*m*/*z*)**	**Ref.**
57	kaempferol-*O*-sinapoylhexosyl-feruloylpentosyl-dicaffeoyl-hexoside	6.30	1447 723 [M−2H]^2−^	**1241** [M−H−206]^−^, **1079** [M−H−206−162]^−^, **771** [M−H−206−162−176−132]^−^, **593** [M−H−206−162−162−162−162]^−^, **285** [M−H−206−162−176−132−162−162−162]^−^ = Y_0_^−^, **284** [Y_0_−H]^−•^, **283** [Y_0_−2H]^−^, **223** [sinapic acid−H]^−^, **205** [sinapoyl−H]^−^, **193** [ferulic acid−H]^−^, **179** [caffeic acid−H]^−^, **175** [feruloyl−H]^−^, **161** [caffeoyl−H]^−^	[23]
67	quercetin-*O*-sinapoyldihexosyl-feruloyldeoxyhexosyl-deoxyhexosyl-hexoside formiate adduct	7.52	1507 753 [M−2H]^2−^	**1255** [M−H−46−206]^−^, **931** [M−H−46−206−162−162]^−^, **609** [M−H−46−206−162−162−176−146]^−^, **301** [M−H−46−206−162−162−176−146−162−146]^−^ = Y_0_^−^, **300** [Y_0_−H]^−•^, **299** [Y_0_−2H]^−^, **205** [sinapoyl−H]^−^, **175** [feruloyl−H]^−^	[23]
**Flavonol-*O*-feruloyl-dihydrophaseoyl-glycosides**					
**No. ^a^**	**Tentative Characterization**	**t_R_ (min) ^a^**	**[M−H]^−^** **(*m*/*z*)**	**Fragment ions** **(*m*/*z*)**	**Ref.**
72	quercetin-3-*O*-dihydrophaseoyl-deoxyhexosyl-feruloylhexosyl-7-*O*-deoxyhexoside	8.05	1195	**1049** [M−H−146]^−^, **931** [M−H−264]^−^, **785** [M−H−146−264]^−^, **609** [M−H−146−264−176]^−^, **301** [M−H−146−264−176−162−146]^−^ = Y_0_^−^, **300** [Y_0_−H]^−•^, **299** [Y_0_−2H]^−^, **175** [feruloyl−H]^−^	[23]
88	kaempferol-3-*O*-dihydrophaseoyl-hexosyl-diferuloylhexosyl-7-*O*-deoxyhexoside	9.85	1371 685 [M−2H]^2−^	**1195** [M−H−176]^−^, **593** [M−H−176−176−162−264]^−^, **285** [M−H−176−176−162−264−162−146]^−^ = Y_0_^−^, **284** [Y_0_−H]^−•^, **283** [Y_0_−2H]^−^,	[23]
91	quercetin-3-*O*-dihydrophaseoyl-deoxyhexosyl-diferuloylhexosyl-7-*O*-deoxyhexoside	10.50	1371 685 [M−2H]^2−^	**1195** [M−H−176]^−^, **1107** [M−H−264]^−^, **609** [M−H−176−586]^−^ = [M−H−176−146−264−176]^−^, **301** [M−H−176−146−264−176−162−146]^−^ = Y_0_^−^, **300** [Y_0_−H]^−•^, **299** [Y_0_−2H]^−^, **193** [ferulic acid−H]^−^, **175** [feruloyl−H]^−^, **145** [coumaroyl−H]^−^	[23]

^a^ Compound numbers and retention times (t_R_) refer to UV chromatograms shown in Figures S1 and S2.

### 2.2. Determination of Minimum Inhibitory Concentration (MIC_80_)

The impact of the leaf (ethanolic and aqueous) extracts of *L. tuberosus* on both Gram-positive and Gram-negative bacteria was assessed by CLSI M07-A9 (Vol. 32, No. 2) guidelines (Table 3). Interestingly, only the ethanolic extract showed an antimicrobial effect, specifically against *Bacillus subtilis* (MIC_80_ value: 354.37 ± 4.58 µg/mL) and *Streptococcus pyogenes* (MIC_80_ value: 488.89 ± 4.75 µg/mL). Neither plant extract (ethanolic and aqueous) had an antibacterial impact on *Escherichia coli*, *Pseudomonas aeruginosa*, or *Staphylococcus aureus*. Erythromycin, as a positive control, has an inhibitory effect on all organisms, as described in Table 3.

### 2.3. Total Antioxidant Capacity (TAC) Assays

**Figure 1:**
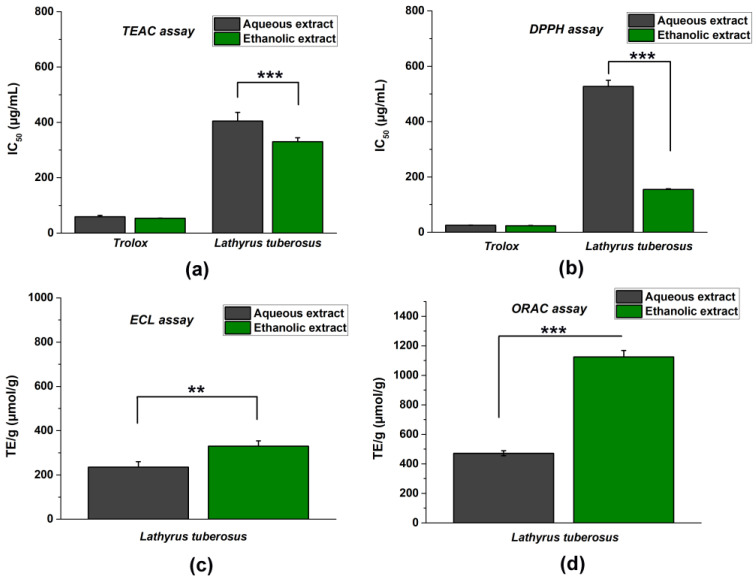
The total antioxidant capacity (TAC) of *L. tuberosus* was assessed using various spectroscopic methods: TEAC assay (**a**); DPPH assay (**b**); ECL assay (**c**); and ORAC assay (**d**). IC_50_ values (representing the concentration at 50% inhibition) were counted for DPPH and TEAC methods, while TE/g values (Trolox equivalent in µmol per 1 g of initial dry material) were determined for ORAC and ECL tests. The comparison between aqueous and ethanolic extracts was evaluated using a t-probe, and the results are presented as mean ± SD from 5 independent experiments, each performed in 3 replicates (** *p* < 0.01, *** *p* < 0.001).

To obtain the most reliable results, we determined the antioxidant characteristics of the ethanolic and aqueous extracts of *L. tuberosus* using approved nonenzymatic chemical methods, including TEAC, DPPH, ECL, and ORAC tests (Figure 1).

It is important to note that since the tests are based on different hydrogen atom transfer (HAT) or single electron transfer (SET) mechanisms, their evaluation was also different. Since the DPPH and TEAC tests are based on endpoint measurement and colour change, the higher antioxidant effect will be indicated by a lower IC_50_ concentration in these inhibitory activity tests. On the contrary, the ECL and ORAC methods are based on kinetic measurements, and therefore, the most robust antioxidant capacity is evidenced by the highest TE/g (μmol/g) concentration values calculated from the area under the curve.

In general, ethanolic extracts demonstrated stronger antioxidant effectiveness compared to aqueous extracts.

### 2.4. Inhibition of Intracellular ROS Production

The determination of the TAC value by cell-free chemical assays does not fully reflect the behavior of complex plant samples in vivo. Therefore, it is important to evaluate the efficacy of antioxidants under more biologically relevant conditions, for example, by testing compounds in cell-based antioxidant assays [54].

The peroxyl radicals generated by AAPH induced the oxidation of DCFH and DHR in keratinocyte cell cultures [55]. Leaf extracts led to a reduction in DCF and derivative rhodamine fluorescence, and the corresponding 50% inhibition values are presented in Table 4.

In general, it can be stated that, as in chemical analyses, the ethanolic extract also exhibited stronger intracellular antioxidant properties in the case of DCFH and DHR tests. Both fluorogenic reporter molecules are sensitive to peroxyl radicals, possessing similar oxidation mechanisms. Therefore, it is not surprising that both DHR and DCFH exhibited similar activity in the presence of plant extracts [55].

### 2.5. Cytotoxicity Test

**Figure 2:**
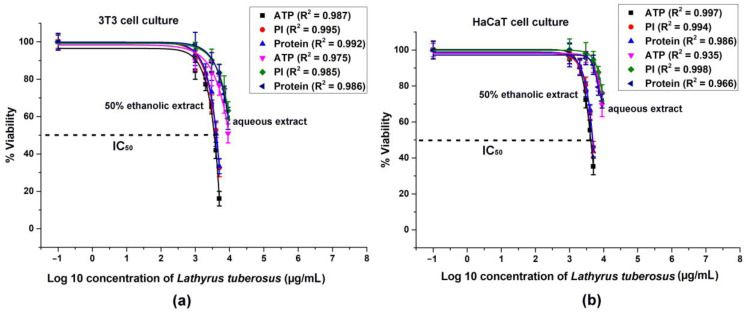
Intracellular ATP level, cell number (PI fluorescence), and total protein content of 3T3 and HaCaT cell culture were analyzed with dose-response fitting, illustrating the cytotoxic effects of *L. tuberosus* on 3T3 (**a**) and HaCaT cells (**b**). The data are presented as a percentage of the control, and the results represent the mean ± SD of 5 independent experiments, with n = 5 × 8 replicates for each concentration. Dose–response curves were generated through log10 transformation and nonlinear curve fitting. Correlation coefficients (R^2^) were calculated for each treatment.

The cytotoxicity data acquired from 3T3 and HaCaT cells is depicted in Figure 2.

A general observation indicates that ethanolic extracts of the plant exhibited greater efficacy in dose-dependent reduction of ATP levels, cell numbers, and total protein contents in both cell lines compared to aqueous extracts. The discrepancy between the solvents could not be attributed solely to the ethanol content, as it was capped at 1.5%, which did not significantly affect cell viability. Consequently, the observed toxic effects were attributed to higher concentrations of active ingredients present in ethanolic extracts.

In the 3T3 cell line, there was a more significant decrease in ATP levels, cell count, and protein concentrations compared to the HaCaT cultures.

### 2.6. In Vitro Migration Test

After evaluating the results of compound analyses and antimicrobial, antioxidant, and cytotoxicity data, we opted to employ the ethanolic extracts exclusively in subsequent experiments. This decision was driven by their more abundant ingredient composition and more potent biological effects. Cell migration investigations were conducted using potentially subtoxic concentrations of the ethanolic extracts (at 50, 100, and 200 g/mL), i.e., where fibroblast and keratinocyte cells showed more than 90% viability by our multiparametric plate-reader assay.

In migration assays, it becomes crucial to discern between cell proliferation and migration since cells are typically not synchronized. Some researchers have addressed this by employing mitomycin C, a DNA synthesis inhibitor, for pretreatment. This approach allows monitoring migration during an extended incubation period (48 h), exceeding the cells’ generation cycle [56]. However, in our experiments, we chose not to utilize this compound due to our limited 24 h incubation time, during which the potential for proliferation was minimal. The HaCaT human keratinocyte cell line exhibits a doubling time of approximately 24 h. In the first passages (2–8), this time extends to 36.2 ± 1.5 h, while in the later passages (10–16), it is reduced to 24 ± 0.6 h [57]. On the other hand, 3T3 mouse fibroblast cells have a doubling time ranging from 20 to 26 h [58].

**Figure 3:**
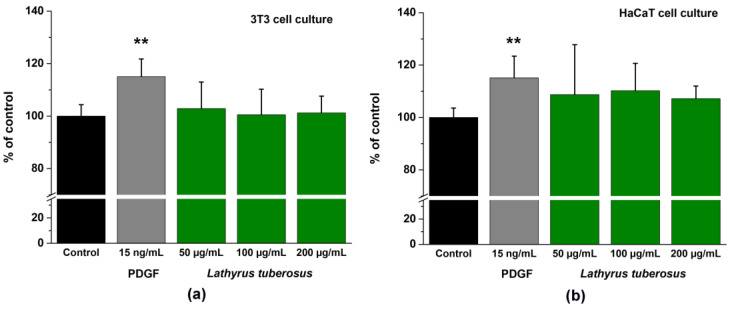
A comparative assessment through time-lapse imaging was conducted to analyze the wound closure capabilities of PDGF-BB and *L. tuberosus* extracts on 3T3 monolayer cultures (**a**) and on HaCaT monolayer cultures (**b**). Cell behavior was observed over a 24-hour period in the absence and presence of plant extract or PDGF, utilizing phase-contrast microscopy. The bar graphs representing the area under the curve (AUC) were calculated based on cumulative closure rates (CR%) assessed at 4-hour intervals across three different concentrations. Results are presented as mean ± SD from three independent experiments, with n = 3 × 3 replicates (** *p* < 0.01 compared to the control).

The application of PDGF-BB as a positive control at a concentration of 15 ng/mL exhibited a noteworthy stimulatory impact on the migration of both cell lines. Specifically, it resulted in a migration increase of 117.05 ± 6.72% for 3T3 cells and 115.16 ± 8.26% for HaCaT cells compared to untreated cells. In contrast, *L. tuberosus* demonstrated a mild stimulating effect, manifesting itself as an elevation of 102.86 ± 9.84% in 3T3 cells and 108.76 ± 10.92% in HaCaT cells at a concentration of 50 μg/mL, as illustrated in Figure 3.

## 3. Discussion

*Lathyrus* species are still an undervalued wellspring of bioactive compounds, especially phenolics, as they have not been explored sufficiently. Our study marks a novel investigation of the biological properties (antioxidant, antimicrobial, cell migration, and cytotoxic effects) and phytochemical profile of *Lathyrus tuberosus*, which was previously ethnomedicinally documented by our research group in Transylvania. We did not find previous data on its antimicrobial, antioxidant, and cell migration effects; therefore, we compared our results based on its active ingredient content or other results from other species of *Lathyrus* species with literature sources. However, the results obtained with literature data are challenging due to several limiting factors. Differences may arise from various factors, including biological aspects such as the drug part of the studied plant, the vegetative phase, and technical considerations such as result evaluation, calibration control, extraction method, solvents, and their concentrations.

Literature data on *L. tuberosus* are scarce. However, the phytochemical composition of other *Lathyrus* species has previously been evaluated [1,21,22,23]. In accordance with these results, in the aqueous and ethanolic extracts of the species, we detected flavonol-*O*-glycosides and acylated flavonol-*O*-glycosides in large numbers, showing extensive diversity. Glycosylated and acylated hydroxycinnamic acid derivatives are present in diverse compositions, too. In addition, *O*- and *C*-glycosides of other classes of flavonoids (e.g., flavones, flavanones, and chalcones), as well as *O*-glycosylated lignans, hydroxybenzoic acids derivatives, and monoterpenes (iridoid and secoiridoid compounds) were also described.

The leaf extract of the plant showed notable antioxidant prowess, mainly the ethanolic extract. Llorent-Martinez et al. tested the antioxidant effects of methanolic extracts of seeds of *L. czeczottianus* Bässler and *L. nissolia* L. using DPPH and TEAC methods. They obtained lower antioxidant properties (DPPH–IC_50_ 1.42 mg/mL and 1.80 mg/mL, TEAC–IC_50_ 1.80 mg/mL and higher than 5 mg/mL). This suggests that the leaf extract we examined has a higher phenolic content, thus exhibiting greater free radical scavenging activity [22].

We demonstrated promising inhibitory effects of *L. tuberosus* against certain Gram-positive bacteria. It is a general observation that Gram-positive bacteria are more susceptible to the inhibitory effects of polyphenols and other antibacterial agents than Gram-negative bacteria, which may be explained by their different cell wall structures [59]. Heydari et al. tested the antimicrobial activity of methanol, n-hexane, ethyl acetate, chloroform, and water fractions of aerial parts of five species of *Lathyrus*, namely *L. armenus* Celak., *L. aureus* (Steven) D. Branza, *L. cilicicus* Hayek & Siehe, and *L. laxiflorus* subsp. *laxiflorus* (Desf.) Kuntze, and *L. pratensis* L. The ethyl acetate fractions of the tested plant species exhibited high antimicrobial activity in the tested microorganisms (*S. aureus*, *B. subtilis*, *E. coli*, *P. aeruginosa*, and *C. albicans*) [60].

On the other hand, the extracts did not show cytotoxic effects on 3T3 fibroblast and HaCaT keratinocyte cell lines at a concentration of 2000 μg/mL for the ethanolic extract and under 5000 μg/mL for the aqueous extract. It is the sensitivity of 3T3 fibroblast cell culture is sensitivity to leaf extract exposure compared to HaCaT keratinocytes. A possible explanation for this might be the location of fibroblasts, whereas keratinocytes are located closer to the epidermis, necessitating increased resilience to external and potentially harmful factors [61].

We did not observe a significant enhancement of cell migration of cells treated by *L. tuberosus*. However, data from the literature demonstrate that hydroxybenzoic acid derivatives measured in the plant extract increase the migration of keratinocytes and fibroblasts [62].

It was a general observation that the ethanolic extract has stronger biological effectiveness (antioxidant, antimicrobial, cytotoxic properties) compared to aqueous extract, which can be attributed to the solvent used in the extraction process. Ethanol is a more efficient solvent for extracting a wider range of antioxidant and antimicrobial compounds, including phenolic compounds, flavonoids, and other polyphenols. Certain properties of bioactive compounds may be more prevalent or exist in more significant concentrations in the ethanolic extract compared to the aqueous extract. Some compounds may be more polar or lipophilic; in this way, ethanol, being a polar solvent with some lipophilic properties, can effectively dissolve and extract a broader spectrum of these compounds compared to water. In contrast, water may not be as effective in extracting certain types of antioxidants due to its polar nature, which limits its ability to dissolve non-polar compounds. On the other hand, caffeic acid-*O*-hexoside, some quercetin-*O*-oligoglycosides, isorhamnetin-*O*-hexosyl-deoxyhexoside, apigenin-7-*O*-sinapoyl-pentoside and monoterpene derivatives were present only in the ethanolic extract, which could cause a higher biological effect.

Our findings propose that deep dives into *Lathyrus* species could pave the way for novel health-promoting agents in the pharmaceutical industries.

## 4. Materials and Methods

### 4.1. Reagents and Chemicals

Ethanol and HPLC grade methanol and acetonitrile were purchased from Molar Chemicals Kft. (Halásztelek, Hungary). Luminol (3-aminophthalhydrazide), 4-iodophenol, Trolox (6-hydroxy-2,5,7,8-tetramethylchroman-2-carboxylic acid), horseradish peroxidase (POD), Na2-fluorescein, AAPH (2,20-azo-bis(2-amidinopropane) dihydrochloride), 2,2-diphenyl-1-picrylhydrazyl (DPPH), potassium persulfate (K_2_S_2_O_8_), 2,20-azino-bis(3-ethylbenzothiazoline-6-sulfonic acid) (ABTS), dichlorofluoresceindiacetate (DCFH-DA), dihydrorhodamine 123 (DHR123), quercetin, modified RPMI 1640 (supplemented with 165 mM MOPS, 100 mM glucose and 0.185 mM adenine), erythromycin, Dulbecco modified Eagle Medium (DMEM), trypsin-EDTA, penicillin–streptomycin for cell culture, propidium iodide (PI) and fluorescamine (Fluram) were acquired from Merck (Darmstadt, Germany). 3-(N-Morpholino) propanesulfonic acid (MOPS) was from Serva Electrophoresis GmbH (Heidelberg, Germany). Ethanol (96% *v*/*v*, spectroscopic grade), glucose, adenine, and hydrogen peroxide (H_2_O_2_) were purchased from Reanal Labour (Budapest, Hungary), while the CLSII bioluminescent ATP Assay Kit and peroxide-free Triton X 100 without peroxide (TX-100) were purchased from Roche (Mannheim, Germany). Foetal bovine serum (FBS; Pan-Biotech, Aidenbach, Germany) and bovine serum albumin (BSA; Biosera, Nuaille, France) were used. The recombinant human platelet-derived growth factor-BB (PDGF-BB) and Hanks’ balanced salt solution (supplemented with 5.5 mM glucose) were from Thermo Fischer Scientific (Waltham, MA, USA). High-purity water (<1.0 µS) was applied throughout the experiments. It was gained by a Millipore Direct Q5 water purification system (Billerica, MA, USA). Plastic cell culture flasks and culture plates (96-well and 24-well) were from TPP (Trasadingen, Switzerland), while standard 96-well plates were from Greiner Bio-One (Kremsmunster, Austria). White 96-well optiplates were used for luminescence studies (Perkin Elmer, Waltham, MA, USA).

### 4.2. Studied Plant Taxa and Plant Extraction

Aerial parts of *L. tuberosus* were collected in Transylvania, Romania, in July 2020. Samples were dried at room temperature and stored in the dark. The voucher specimen of the plant labelled with a unique code was deposited at the Department of Pharmacognosy, University of Pécs, Pécs, Hungary (Voucher code: HV_10). The plant name follows the terminology of The World Flora Online (WFO, 2023).

For UHLC measurements, the aqueous and 50% (*v*/*v*) ethanolic extracts of the leaf were obtained by extracting 3.0 g of leaf powder in 30 mL of distilled water or 50% (*v*/*v*) ethanol using an ultrasonic bath (Bandelin Sonorex Digitec DT 1028, Berlin, Germany) three times, 30 min each (nominal ultrasonic power: 300 W, ultrasound frequency: 35 kHz). The extracts were distilled to dryness under reduced pressure with a rotary evaporator (Büchi Rotavapor R-200, Flawil, Switzerland) at 45 °C. The residues were dissolved in 20 mL of 70% (*v*/*v*) HPLC grade methanol and filtered through Minisart RC 15 0.2 µm syringe filters (Sartorius AG, Goettingen, Germany).

For antioxidant, antimicrobial and cellular measurements, plant extraction was conducted following our previous methodology without modifications [3].

### 4.3. Analyses of Phenolic Compounds by Ultrahigh-Performance Liquid Chromatography (UHPLC) Coupled to Diode-Array Detector (DAD) and Mass Spectrometry (MS)

#### 4.3.1. UHPLC Conditions

The chromatographic separation was performed on an ACQUITY™ UPLC™ H-Class PLUS System (Waters Corporation, Milford, MA, USA). Samples were separated on an Acquity UPLC BEH C18 (Waters, Dublin, Ireland) (100 mm length, 2.1 mm i.d., 1.7 µm particle diameter) column, maintained at 30 °C. The mobile phase was composed of 0.3% (*v*/*v*) acetic acid in water (A) and acetonitrile (B). All aqueous solvents were filtered through membrane filters of MF-Millipore (Millipore, Billerica, MA, USA) (0.45 µm, mixed cellulose esters). The following gradient elution was applied at a flow rate of 0.3 mL/min: 0.0 min 15% B, 10.0 min 25% B, 16.0 min 80% B, 16.5 min 100% B, 19.0 min 100% B. UV spectra and chromatograms were recorded at 200–400 nm. The injection volume was 5 µL.

#### 4.3.2. MS Conditions

Mass spectrometric analyses were performed with a Xevo Q-TOF instrument equipped with an electrospray ionisation source (ESI) (Waters Corporation). ESI conditions were as follows: capillary voltage 2.6 kV, sampling cone voltage 40 V, source temperature 120 °C, desolvation temperature 300 °C, desolvation N_2_ gas flow 600 L/h, collision energy was changed between 10 eV and 45 eV, depending on the structure analyzed. High purity nitrogen was used as the collision gas. Full-scan mass spectra were acquired in the range of *m*/*z* 100–2000 in negative ionisation mode. Masslynx 4.1 software was used for data acquisition and qualitative analysis.

### 4.4. Determination of Minimum Inhibitory Concentration (MIC_80_) with Microdilution Method

All bacterial strains were sourced from the Szeged Microbiology Collection (SZMC), Department of Microbiology, University of Szeged, Hungary, and the Pécs Microbiology Collection (PMC), Department of General and Environmental Microbiology, Institute of Biology, University of Pécs, Hungary. The strains under investigation included *Bacillus subtilis* (*B. subtilis*, SZMC strain: 0209), *Escherichia coli* (*E. coli*, PMC strain: 201), *Staphylococcus aureus* (*S. aureus*, ATCC strain: 29213), *Streptococcus pyogenes* (*S. pyogenes*, SZMC strain: 0119), and *Pseudomonas aeruginosa* (*P. aeruginosa*, PMC strain: 103).

The microdilution method was performed in accordance with a previously published protocol with slight modifications [63]. In summary, 100 µL of bacterial suspensions (10^5^ CFU/mL) in RPMI 1640 media and 100 µL of diluted aqueous or 50% (*v*/*v*) ethanolic leaf extracts in media were dispersed in each well of sterile 96-well plates. The negative control consisted of sterile medium, the bacterial growth control involved RPMI 1640 without treatment, and erythromycin served as a positive control. The ethanolic solvent concentration for dilution was capped at 1.0% (*v*/*v*) in the wells. Absorbance readings at 595 nm were recorded using a Multiskan EX 355 (Thermo Electron Corporation, Waltham, MA, USA) spectrophotometer after 24 h of incubation at 30 °C. Absorbance values below 20% of bacterial growth controls were designated as MIC_80_.

### 4.5. Total Antioxidant Capacity (TAC) Assays

#### 4.5.1. Oxygen Radical Absorbance Capacity (ORAC) Assay

The ORAC test was implemented following the methodology of Kőszegi et al. without any alterations [64]. Serial dilutions were employed as the standard. In summary, 150 µL of working fluorescein solution (400 nM dissolved in 75 mM potassium phosphate buffer, pH 7.5) and 25 µL of blank/standard/plant extract (aqueous/ethanolic) were distributed in each well of standard 96-well plates. The plates underwent a preincubation period of 30 min at 37 °C in the dark. Subsequently, 25 µL of AAPH solution (400 mM dissolved in 75 mM potassium phosphate buffer) was automatically injected and fluorescence intensities were measured in kinetic mode for 80 min at 37 °C, with excitation and emission wavelengths set at 490 and 520 nm, respectively. A plate reader (BioTek Synergy HT, Winooski, VT, USA) was used for measurements.

#### 4.5.2. Enhanced Chemiluminescence (ECL) Assay

This method was carried out according to our previously published study, without any alterations [64]. In summary, a premixture of 70 µL of ECL detection reagent (0.15 M boric acid/NaOH, pH 9.6, supplemented with 0.45 mM luminol and 1.8 mM 4-iodophenol) and 200 µL POD enzyme solution (15 µU/mL) was maintained on ice. Trolox dilutions served as the standard. In each well of white optical 96-well plates, 20 µL of Trolox/blank/sample and 270 µL of POD-ECL reagent were introduced. The reaction started with the automated injection of 20 µL ice-cold H_2_O_2_ (1.5 mM, 0.1% citric acid). The chemiluminescence signal was observed for 10 min using a plate reader (Biotek Synergy HT) in kinetic analysis mode.

#### 4.5.3. Radical Scavenging Assay for 2,2-Diphenyl-1-picrylhydrazyl (DPPH)

The measurement procedure was carried out according to a previously described protocol [65,66], with some modifications. In summary, 50 µL of blank/standard/plant sample dilutions were followed by the addition of 100 µL of DPPH (200 µM, dissolved in 96% ethanol) and 50 µL of acetate buffer (100 mM, pH 5.5) in general microplates. Absorbance changes were monitored at 517 nm using a Perkin Elmer EnSpire Multimode plate reader (Perkin Elmer, Waltham, MA, USA) after 60 min of incubation in the dark at room temperature. The obtained results were compared to serial dilutions of a Trolox standard solution.

#### 4.5.4. Trolox Equivalent Antioxidant Capacity Assay (TEAC)

The TEAC test was conducted with a slight modification of the approach outlined by Re et al. and Stratil et al. [67,68]. ABTS^•+^ was generated by reacting the ABTS stock solution (7 mM of ABTS dissolved in distilled water) with 2.45 mM K_2_S_2_O_8_ (final concentration) and diluting the mixture with PBS (pH 7.4) until the absorbance reached 0.70 ± 0.005 at 734 nm. Subsequently, 20 µL aliquots of various concentrations of leaf extracts (ethanolic/aqueous) were allowed to react with 80 µL of ABTS^•+^ (7 mM), and absorbance readings were recorded at 734 nm using the Perkin Elmer plate reader after a 20-minute incubation in the dark at room temperature. Trolox served as the standard.

#### 4.5.5. Calculation of Total Antioxidant Capacities (TAC)

For the ORAC and ECL assays, the outcomes were determined in terms of Trolox equivalents (TE). In the ORAC method, the net area under the fluorescence curve (netAUC) was calculated by subtracting the AUC of the blank from that of the standard/sample. Then a calibration line was established for the netAUC of Trolox standards. In the ECL technique, the AUC of the emission curves against Trolox standards was utilized to derive the calibration line. In both scenarios, the TE values of the samples were derived from the calibration curves, which were subsequently adjusted by the dilution factor and expressed as µM TE concentration. Ultimately, the total antioxidant capacity (TAC) was normalized to 1 g of initial dry material for each plant sample.

In both the DPPH and TEAC assays, radical scavenging activity was denoted as IC_50_, representing the concentration of plant extract in µg/mL needed to scavenge 50% of the reactions of DPPH or ABTS. This was calculated using a linear regression curve established from the scavenging activities versus the amount of extracts in the samples. Consequently, a lower IC_50_ value indicates a higher antioxidant activity in the sample.

Calculating the radical scavenging activity for the leaf extracts, presented as a percentage relative to the blank, was carried out using the following formula:

(1) $$Radical\;scavenging\;activity\left(\%\;inhibition\right)=\left(\frac{A_{0}-A_{1}}{A_{0}}\right)\times 100$$

where *A*_0_ is the absorbance of the blank and *A*_1_ is the absorbance of the sample.

### 4.6. Cell Cultures

Mouse fibroblasts (3T3, ATCC: CRL-1658) were grown in DMEM with high glucose (4500 mg/L), supplemented with 5% non-essential amino acids, 10% FBS, penicillin (100 U/mL) and streptomycin (100 µg/mL). Meanwhile, the immortalised human epidermal keratinocyte cell line (HaCaT), generously provided by the laboratory of Prof. Tamás Boró (Department of Immunology, Faculty of Medicine, University of Debrecen, Hungary), was cultured in DMEM with high glucose (4500 mg/L), supplemented with 10% FBS, penicillin (100 U/mL), and streptomycin (100 µg/mL) in cell culture flasks at 37 C in a humidified atmosphere with 5% CO_2_.

### 4.7. Quantification of Intracellular ROS

To evaluate cellular oxidative stress induced by excessive generation of reactive oxygen species (ROS) by AAPH, we employed the DCFH-DA and DHR123 methods [69,70,71]. The optimal conditions for conducting the DCFH-DA and DHR123 assays on 96-well culture plates involved a seeding density of 5 × 10^4^ cells/mL and an overnight incubation of the cell culture. Following a PBS wash, co-incubation was carried out in Hanks solution (5.5 mM glucose) with 50 μM DCFH-DA or 10 μM DHR123, in the presence of plant extract/quercetin/Trolox on 3T3/HaCaT cells, for 1 h. Upon removal of the treating medium and a subsequent washing with PBS, 1 mM AAPH oxidant was introduced in Hanks glucose. The fluorescence intensity was monitored for 60 min using the Biotek microplate reader at 490/520 nm excitation/emission wavelengths, maintaining a temperature of 37 °C. The determination of radical scavenging activity involved quantifying the IC_50_, which represents the concentration (in µg/mL) of the plant sample required to scavenge 50% of the DCFH or DHR123 fluorescence. This was achieved by linear regression analysis of serial dilutions of leaf extracts. The expression of radical scavenging activity was computed using the provided equation.

(2) $$Radical\;scavenging\;activity\left(\%\;inhibition\right)=\left(\frac{{AUC}_{0}-{AUC}_{1}}{{AUC}_{0}}\right)\times 100$$

where AUC_0_ refers to the area under the curve values for the blank, while AUC_1_ corresponds to the area under the curve values for the sample. Each treatment was subjected to four technical replicates during five independent experiments.

### 4.8. Cytotoxicity Test

We conducted a multiparametric viability assay with one-step extraction, following the methodology outlined in our previously published study, without any modifications [72]. The aim was to assess the potential toxicity of 50% (*v*/*v*) ethanolic and aqueous leaf extracts. of *L. tuberosus.* The extracts were tested at concentrations ranging from 100 to 5000 µg/mL (ethanolic) and 1000 to 9000 µg/mL (aqueous). The concentration of ethanolic solvent was capped at 1.5% (*v*/*v*) in the wells, a level that does not affect cell viability. In summary, 3T3 and HaCaT cells were exposed to various concentrations of plant extracts for 24 h. Following treatment, ATP levels were measured from cell lysates using the bioluminescence method. The nucleic acid content was analyzed through PI staining, while total levels of intracellular protein were quantified after fluorescent derivatization with fluorescamine. The dose–response curves were calculated from the measured data using OriginLab Pro software (version 2016, OriginLab Corporation, Northampton, MA, USA) after the DoseResp fitting.

### 4.9. In Vitro ‘Wound Healing’ Assay

To test cellular migration, we used our previous method without any modifications [3]. The ethanolic plant extract was tested at subtoxic concentrations of 50, 100, and 200 µg/mL. PDGF-BB served as a positive control at a concentration of 15 ng/mL. Cell migration within the cell-free gap was visualized every 4 h for 24 h by time-lapse imaging in bright field, utilizing phase-contrast microscopy (JuLi Stage Real-Time Cell History Recorder, NanoEnTek, Seoul, Republic of Korea). The gap was monitored at an objective magnification of 10×. The closed cell-free area closure rate was determined by quantifying the microphoto density data obtained for each occasion from the same locations using ImageJ 1.x processing software (https://imagej.nih.gov/ij/ accessed on 11 December 2023). The closure rate, expressed as a percentage, was calculated using the provided formula.

(3) $$Closure\;rate\left(\%\right)=\left(\frac{{Open\;area}_{0.h}-{Open\;area}_{x.h}}{{Open\;area}_{0.h}}\right)\times 100$$

The “open area 0.h” represents the initial cell-free area at the start of the experiment, while the “open area x.h” represents the remaining cell-free space at various time points during sample imaging. Subsequently, closure rate curves were generated, and the area under the curve (AUC) was calculated for each treatment and cell line. The AUC data was then averaged (±SD), and the summarised closure rates for the leaf extracts/PDGF were presented as a percentage (%) relative to the untreated controls.

### 4.10. Statistical Analyses

If applicable, data were presented as a percentage relative to control samples, assumed to be around 100%. In the cytotoxicity test, correlation coefficients for each parameter tested were provided for the dose–response curves. Statistical analysis for the migration assay involved the use of a one-way analysis of variance (ANOVA) test, comparing control and sample data for a specific treatment through SPSS software (IBM, SPSS Statistics, version 22, Armonk, NY, USA). In all cases, the significance level was set at *p* < 0.05.

## 5. Conclusions

Medicinal plants play an important role in ethnomedicine as well as in our research carried out in Transylvania, Romania. In this work, the detailed polyphenolic contents of *Lathyrus tuberosus* and their related antioxidant, antimicrobial, cytotoxic, and cell migration activities were demonstrated for the first time. Considerable biological abilities of ethanolic and aqueous extract of *L. tuberosus* are mainly attributed to the high level of polyphenolic substances, such as O- and C-glycosides of flavonoids as well as O-glycosylated lignans, hydroxybenzoic acid derivatives, and monoterpenes. A comprehensive characterisation of 93 compounds was accomplished through UHPLC-MS/MS. Our results provide evidence that the tested plant is capable of directly quenching free radicals to terminate the radical chain reaction, acting as a reducing agent. The ethanolic extract exhibited noteworthy antimicrobial activity by inhibiting the growth of *Bacillus subtilis* and *Streptococcus pyogenes*, as demonstrated in the microdulution method. The outcomes highlighted in this paper have inspired our next study for isolation and structure clarification of the active constituents of *L. tuberosus*.

## Figures and Tables

**Table 3:** Minimum inhibitory concentration (MIC_80_) of *L. tuberosus* extracts on *S. aureus*, *B. subtilis*, *S. pyogenes*, *E. coli*, and *P. aeruginosa*.

	MIC80 [µg/mL]		
Test Bacteria	Ethanolic Extracts of *L. tuberosus*	Aqueous Extracts of *L. tuberosus*	Erythromycin ^a^
*Staphylococcus aureus*	N.D.	N.D.	0.16 ± 0.07
*Bacillus subtilis*	354.37 ± 4.58	N.D.	0.13 ± 0.04
*Streptococcus pyogenes*	488.89 ± 4.75	N.D.	0.08 ± 0.01
*Escherichia coli*	N.D.	N.D.	37.13 ± 4.95
*Pseudomonas aeruginosa*	N.D.	N.D.	79.85 ± 9.93

Mean ± SD of 5 independent experiments, each in 3 replicates (n = 5 × 3) compared with erythromycin as positive control (^a^); N.D.: not detectable.

**Table 4:** The cell-based antioxidant capacity of *L. tuberosus* extracts was assessed using DCFH-DA and DHR123 on 3T3 and HaCaT cells. The calculation of the 50% inhibitory concentrations was conducted utilizing the equations derived from the inhibitory capacities observed in the serial dilutions of the extracts.

	IC_50_ [µg/mL]			
	DCFH-DA		DHR123	
	Ethanolic Extr.	Aqueous Extr.	Ethanolic Extr.	Aqueous Extr.
*L. tuberosus* effects on 3T3 cells	389.78 ± 49.93	1664.29 ± 266.96	62.37 ± 8.24	89.11 ± 14.72
*L. tuberosus* effects on HaCaT cells	959.60 ± 59.67	5160.07 ± 349.51	65.70 ± 8.97	88.03 ± 11.67

Mean ± SD of 5 independent experiments, n = 5 × 4 replicates for each concentration.

## Data Availability

The data presented in this study are available on request from the corresponding author.

## Supplementary Materials

The following supporting information can be downloaded at https://www.mdpi.com/article/10.3390/plants13020232/s1: Figure S1: UHPLC-DAD chromatogram of *L. tuberosus* 50% (*v*/*v*) ethanolic extract. Detection wavelength: 320 nm. Numbering of peaks refers to data shown in Table 1 and Table 2. Figure S2: UHPLC-DAD chromatogram of *L. tuberosus* aqueous extract. Detection wavelength: 320 nm. Numbering of peaks refers to data shown in Table 1 and Table 2.
