# Supplementary figures and images for: A Comprehensive Study on Lathyrus tuberosus L.: Insights into Phytochemical Composition, Antimicrobial Activity, Antioxidant Capacity, Cytotoxic, and Cell Migration Effects
Source: Plants (Basel). 2024 Jan 14;13(2):232. doi: 10.3390/plants13020232 (PMC10821300; doi:10.3390/plants13020232)

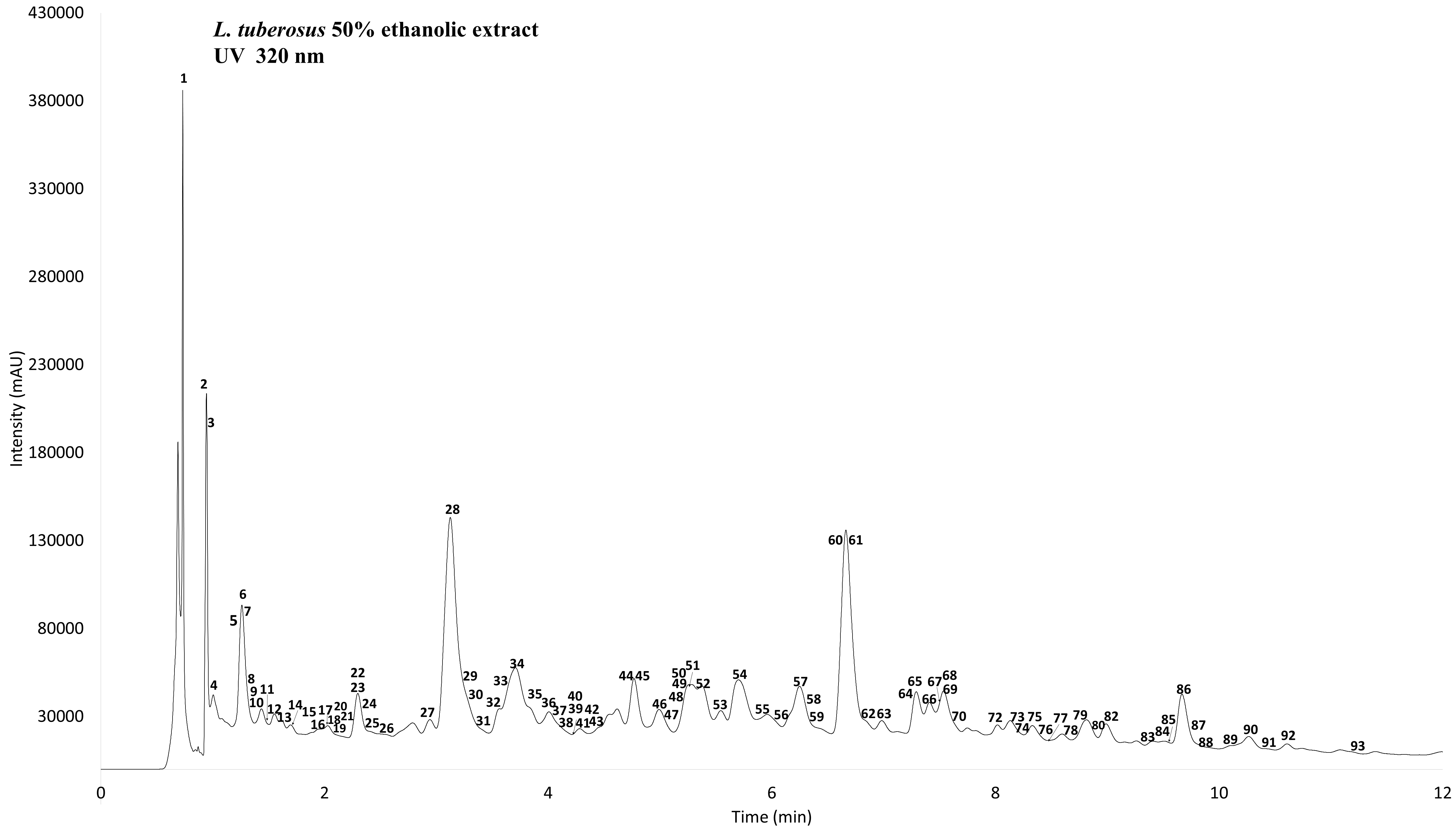

Supplement: Supplementary file 1 [file plants-13-00232-s001.zip › Jakabfi-Csepregi_Lathyrus_Figure S1.TIF]

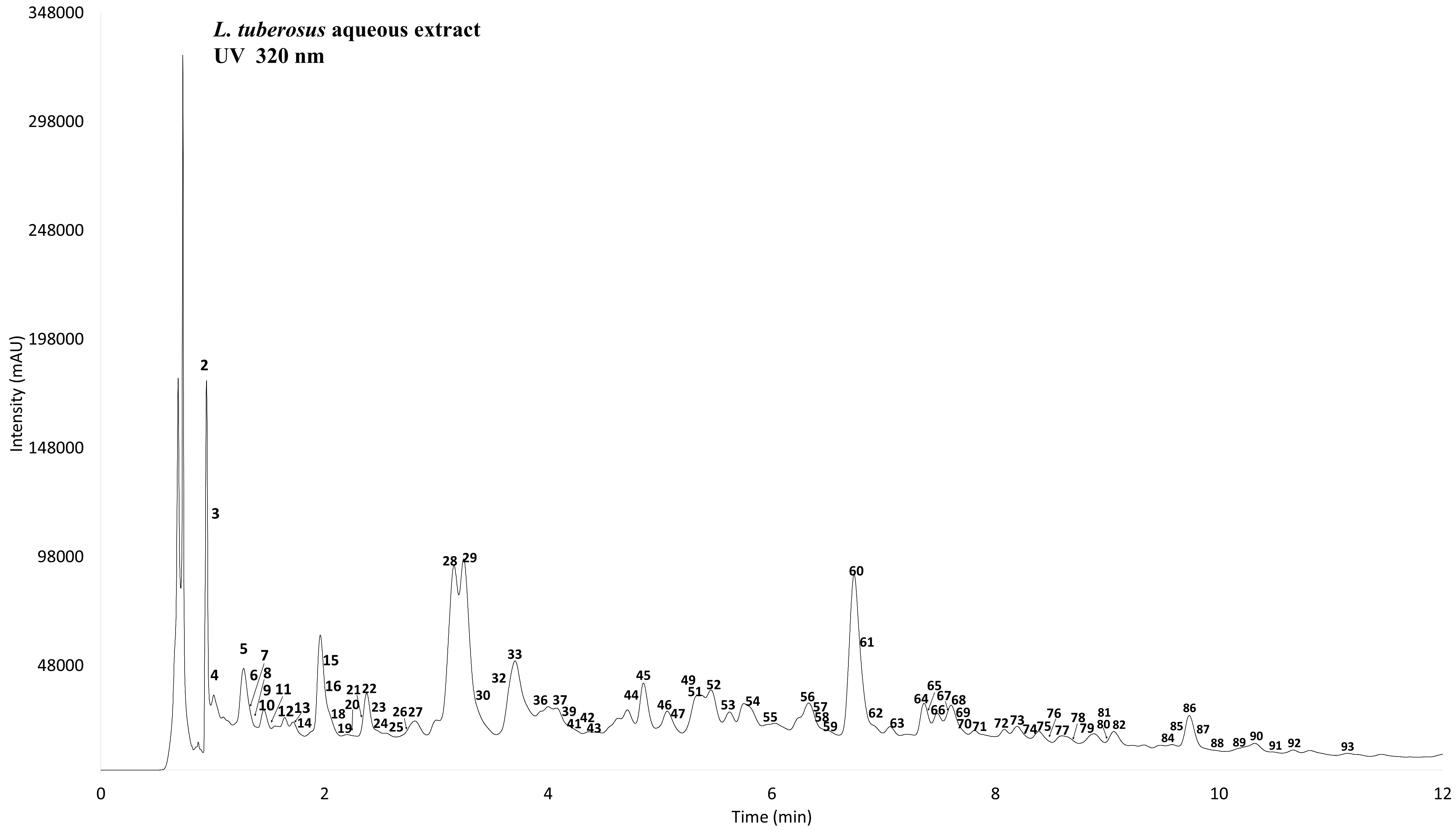

Supplement: Supplementary file 1 [file plants-13-00232-s001.zip › Jakabfi-Csepregi_Lathyrus_Figure S2.TIF]
